# Childhood socio‐economic disadvantage predicts reduced myelin growth across adolescence and young adulthood

**DOI:** 10.1002/hbm.25024

**Published:** 2020-05-20

**Authors:** Gabriel Ziegler, Michael Moutoussis, Tobias U. Hauser, Pasco Fearon, Edward T. Bullmore, Ian M. Goodyer, Peter Fonagy, Peter B. Jones, Ulman Lindenberger, Raymond J. Dolan

**Affiliations:** ^1^ Max Planck University College London Centre for Computational Psychiatry and Ageing Research University College London London UK; ^2^ Max Planck University College London Centre for Computational Psychiatry and Ageing Research Max Planck Institute for Human Development Berlin Germany; ^3^ Wellcome Centre for Human Neuroimaging University College London London UK; ^4^ Institute of Cognitive Neurology and Dementia Research Otto‐von‐Guericke‐University Magdeburg Magdeburg Germany; ^5^ DZNE Magdeburg German Center for Neurodegenerative Diseases Magdeburg Germany; ^6^ Department of Psychiatry University of Cambridge Cambridge UK; ^7^ Research and Development Department Cambridgeshire and Peterborough National Health Service Foundation Trust Cambridge UK; ^8^ Medical Research Council/Wellcome Trust Behavioural and Clinical Neuroscience Institute University of Cambridge Cambridge UK; ^9^ Research Department of Clinical, Educational and Health Psychology University College London London UK; ^10^ Center for Lifespan Psychology Max Planck Institute for Human Development Berlin Germany

**Keywords:** adolescence, development, longitudinal, magnetization transfer, myelin, parental education, parenting, quantitative imaging, socio‐economic disadvantage, young adulthood

## Abstract

Socio‐economic disadvantage increases exposure to life stressors. Animal research suggests early life stressors impact later neurodevelopment, including myelin developmental growth. To determine how early life disadvantage may affect myelin growth in adolescence and young adulthood, we analysed data from an accelerated longitudinal neuroimaging study measuring magnetisation transfer (MT), a myelin‐sensitive marker, in 288 participants (149 female) between 14 and 25 years of age at baseline. We found that early life economic disadvantage before age 12, measured by a neighbourhood poverty index, was associated with slower myelin growth. This association was observed for magnetization transfer in cortical, subcortical and core white matter regions, and also in key subcortical nuclei. Participant IQ at baseline, alcohol use, body mass index, parental occupation and self‐reported parenting quality did not account for these effects, but parental education did so partially. Specifically, positive parenting moderated the effect of socio‐economic disadvantage in a protective manner. Thus, early socioeconomic disadvantage appears to alter myelin growth across adolescence. This finding has potential translational implications, including clarifying whether reducing socio‐economic disadvantage during childhood, and increasing parental education and positive parenting, promote normal trajectories of brain development in economically disadvantaged contexts.

## 1 INTRODUCTION

Socio‐economic disadvantage (SED) is associated with increased exposure to childhood adversity (Pascoe et al., 2016), and is also associated with problematic physical and mental health and poorer educational and employment outcomes (Evans & Cassells, 2014; Johnson, Riis, & Noble, 2016; McDermott et al., 2019). The impact of early life socio‐economic disadvantage (SED) on brain development is poorly understood in terms of fine structure of the brain. Here, we avail of a unique longitudinal sample of young people and in vivo quantitative magnetic resonance imaging (MRI) that provides a measure of macromolecular content sensitive to myelin, to examine the effects of SED on myelin development.

Animal studies, where early stressors are under experimental control, show a causal impact of adversity on brain growth (Howell et al., 2013; Liu et al., 2012; Zhang, 2017). Although they cannot be generalised directly to human populations, animal studies are critical for pinpointing the consequences of risk exposure to processes such as myelination, which are measurable in humans. Here microstructural measures are mechanistically important, as cortical myelin likely reflects local neuritic insulation and fibre density (Glasser, Goyal, Preuss, Raichle, & Van Essen, 2014). It also enables myeloarchitectonic parcellation (Glasser et al., 2016) and influences neuronal dynamics (Demirtas et al., 2019). Understanding mechanisms can improve conclusions about causality (Broadbent, 2011), and weaknesses in causal claims (Wax, 2017) can be bolstered by studying critical markers of brain development in relation to SED exposure (Donahue, Glasser, Preuss, Rilling, & Van Essen, 2018).

We recently mapped neurotypical myelin development during adolescence and young adulthood, using myelin‐sensitive magnetization transfer saturation (MT) (see also (Turati et al., 2015) and we showed that myelin growth is tied to aspects of mental health (Ziegler et al., 2019). It is important to extend this approach to studying the possible impact of SED. Early life adversity has many components pertinent to SED, and with respect to brain development, adversity has recently characterised in terms of two dimensions, 'threat' and 'deprivation'. Threat, such as exposure to violence, is related to reduced grey matter thickness or volume of medial prefrontal and medial temporal structures (Butler, Yang, Laube, Kühn, & Immordino‐Yang, 2018; Saxbe et al., 2018). In contrast, deprivation is related to reductions in dorsolateral prefrontal and superior parietal thickness or volume, with some evidence also for reduced fractional anisotropy in frontoparietal tracts (McLaughlin, Weissman, & Bitran, 2019). Studies have thus linked structural brain measures to early life adversities that are commoner in the presence of SED, as well as to SED as a whole (McDermott et al., 2019; Noble et al., 2015), yet the contribution to these findings of alterations in myelin development is uncertain.

Additionally, studies tentatively implicate myelin in the impact of deprivation. For example, childhood cortisol reactivity to stress has been linked to reduced fractional anisotropy (Sheikh et al., 2014), while in young adulthood developmental stressors are linked to altered myelin measures (Jensen et al., 2017). Here, increased or decreased measures of myelination are reported that is dependent on the developmental timing of the associated stressors. These findings beg the question as to how myelination, across the entire brain, unfolds during adolescence, in relation to early socio‐economic disadvantage rather than as a function of specific stressors.

In this study, we asked whether early SED is associated with a distinct pattern of longitudinal myelin growth during late adolescent and early adult development. We hypothesised that SED would impact brain development, specifically predicting that neighbourhood‐level indices of deprivation would be associated with alterations in both mean level of myelination, adjusting for age (MT_mean_), and the rate of myelin growth (MT_rate_) during adolescent brain development. Following up on several recent studies, we tested whether parental education and family income are critical features of the impact of SED on brain development (Hanson, Chandra, Wolfe, & Pollak, 2011), and whether parenting quality might potentially mediate or moderate the relationship between SED and myelination (Luby et al., 2013). We explored whether key individual characteristics might explain relationships between the environment and brain development. Importantly, both SED and poor parenting (Sleddens, Gerards, Thijs, De Vries, & Kremers, 2011) increase the risk of being overweight (Salmasi & Celidoni, 2017), which in turn is associated with deviant white matter development (Kullmann, Schweizer, Veit, Fritsche, & Preissl, 2015). Finally, differences in general intelligence are a crucial correlate of anatomical brain features that depend on SED (McDermott et al., 2019). Therefore we examined whether body mass index (BMI) and general intelligence (IQ) accounted statistically for any relationships between SED and myelination.

## 2 MATERIALS AND METHODS

### 2.1 Recruitment, demographic and psychological measures

Participants were recruited from the Neuroscience in Psychiatry network participant pool (Kiddle et al., 2017). From this pool, also known as the '2K sample', 300 participants were recruited for the present scanning study. We aimed to exclude all but the most minor psychiatric and neurological symptomatology, and therefore screened participants by self‐report and Structured Clinical Interview for DSM‐5(SCID) to not have current or previous relevant medical histories (First, 2015). Our recruited sample had *N* = 300 and was balanced for sex. At baseline, scanned participants had median age 18.7 years (range 14.1–25.0; interquartile 4.76 yr) and at follow‐up a median of 19.6 years (range 15.11–26.3; interquartile 4.73 yr; Figure S1).

The age range, size and follow‐up intervals employed were determined on the basis of previous studies (Giedd, 2004) but also formal power analyses. The reader is referred to part A of the Data S1 for more detail. The power‐analytic process resulted in a decision to recruit 300 participants in 5 equally spaced two‐year bins, matched for sex, and a preferred inter‐scan interval of 12–18 months. Twenty‐six participants were also invited for a shorter, 6‐month follow‐up scan.

We analysed 497 available brain scans in all obtained from 288 healthy (149 female) individuals that passed quality control. In total, quality control resulted in exclusion of 61 out of 558 originally available scans, resulting in 497 scans entering analysis. 30 out of 318 participants had at least one excluded scan, including 12 who provided no scans of adequate quality. In particular, data from 100, 167, and 21 subjects with one, two or three visits per person were available, with mean (standard deviation) follow‐up interval of 1.3 (0.32) years between first and last visit (see Figure S1). The numbers of follow‐ups were not associated to the age at baseline (*r* = .0183, *p* = .76).

Self‐defined race group was asked about shortly after recruitment and in terms of the following groups: White (245, or 85% of declared race), Black (6, or 2%), Asian (11, or 3.8%) Mixed (20, or 6.9%), Other (4, or 1.4%), 'Prefer not to say' (3, or 1%). On the day of scanning, participants also completed the Wechsler Abbreviated Scale Intelligence of (WASI) (Kiddle et al., 2017). As ours was a developmental study, we used the raw subscale scores for vocabulary and matrix IQ and explicitly analysed their dependence of age. Unless otherwise stated, IQ measurements refer to the time of the first, 'baseline' scan.

We sought to examine the role of parental caregiving quality, as experienced by young people themselves. We collected and analysed self‐report questionnaires from all our scanned participants, as well as the large cohort from which they were sampled, and from this derived a measure of overall parenting quality. We formed a composite score of the Positive Parenting Questionnaire (PPQ), Alabama Parenting Questionnaire (APQ) and Measure of Parenting Style (MOPS). All three were obtained within about a month of the first scan (Kiddle et al., 2017). We took the reversed positive parenting total score from the PPQ and the similarly reversed positive parenting scales from the APQ, the negative parenting scales from the APQ (inconsistent discipline, poor supervision, and corporal punishment) and the negative parenting scores for the MOPS (abuse, control and neglect). These were standardised and summed to make a composite negative parenting scale. In summary, we subtracted all negative items scores from all positive item scores, as positive and negative items were highly anticorrelated, to derive the overall score. The internal consistency of the resulting total score was alpha = .96.

As far as parental education and all other socio‐economic measures are concerned, either parents (for participants younger than 18) or participants themselves (if over 18) reported the highest qualification and occupational level of the parents (Kiddle et al., 2017). This data was obtained for the mother, the father, and if applicable the mother's partner and the father's partner. These were converted to an ordinal scale, according to a categorisation of educational achievement in England—that is, none, primary school, secondary school—GCSE's, sixth form—A levels, skills‐based trainings, undergraduate education, postgraduate education or higher professional training. We then took as starting score the education level of the female parent (usually biological mother) and compared it with the primary male parent (mother's partner or biological father, in that order of priority). It was unusual for these to differ by more than one on this ordinal scale. Therefore, if only one parent score was available, we used that for 'parental education'; otherwise we averaged the two prioritised scores.

### 2.2 Measures of socio‐economic disadvantage

We used the following measures of socio‐economic disadvantage. As our central measure of SED, we used the neighbourhood proportion of households below the official poverty income around the participant's residence (Fry, 2010) at the time of first scan. In addition to SED based on their current neighbourhood, participants were asked in addition to provide the principal address where they resided before 12 years of age. We also used an index of parental education (IPE); and the mother's and father's SOC2000 occupational class (HESA, 2003).

All non‐imaging study data were collected and managed using REDCap electronic data capture tools hosted at the University of Cambridge and at University College London (Harris et al., 2009). REDCap (Research Electronic Data Capture) is a secure, web‐based application designed to support data capture, storage, processing and download for research studies.

### 2.3 MRI data acquisition and longitudinal preprocessing

We focused on magnetisation transfer saturation (MT) rather than morphometric indices as our primary measure of (myelin) development, following priorities highlighted recently by neurodevelopmental researchers (Walhovd, Fjell, Giedd, Dale, & Brown, 2017). We analysed grey and white matter MT changes related to age, both within and across participants, using the efficient 'sandwich estimator' (Guillaume et al., 2014) while adjusting for curvilinear trajectories. We now describe the experimental process in more detail (but see also the Data S1).

Brain scans were acquired using a multi‐echo, fast‐low‐angle‐shot, multiparameter mapping protocol (FLASH MPM; for further details see supplemental methods in Data S1) in 3T Siemens Magnetom TIM Trio systems located in Cambridge (two sites) and London (one site). Reproducibility of maps within this study was assessed in (Weiskopf et al., 2013) and scanner effects were accounted for by adding a covariates in our analyses. Isotropic 1 mm MT saturation maps were collected to quantify local myelin‐related changes throughout the brain. Analyses were performed using SPM12 (Wellcome Centre for Human Neuroimaging, London, UK, http://www.fil.ion.ucl.ac.uk/spm), the h‐MRI toolbox for SPM (Tabelow et al., 2019) (https://github.molgen.mpg.de/hMRI-group/Toolbox), Computational Anatomy toolbox (CAT12, http://www.neuro.uni-jena.de/cat/) and the tools described in more detail below and in Section 2 of the preceding paper that focussed on effects of demographics in the same sample (Ziegler et al., 2019).

To assess macromolecular growth during development, we used a longitudinal Voxel‐Based Quantification (VBQ) pipeline that follows the following steps (for more details and illustration see Ziegler et al., 2019). First, images were serially registered. Each baseline—follow‐up mid‐point image was then segmented into grey matter (GM), white matter (WM) and cerebrospinal fluid, using the CAT12 toolbox of SPM. MT maps from all time‐points were then normalised to MNI space, manually inspected and checked for outliers.

In order to avoid motion‐induced artefacts, we discarded the 10% of images with the strongest artefacts, and also included a motion covariate in analyses according to the following rationale. Recent work (Castella et al., 2018) studied prospective motion correction in the context of Multi Parameter Mapping (MPM). During MPM generation a multi‐echo model was estimated. The standard deviation parameter of R2* residuals in white matter areas (denoted as SDR2*) has been shown to be an accurate proxy of an individual's movement during a scan. This during scan motion proxy was assessed to discard 10% of images with strongest motion‐induced artefacts. Moreover, to control for residual effects of motion‐induced variability, we included SDR2* in all MT analyses as a confounding variable, rendering the presented associations linearly independent of this proxy of absolute motion. We constructed masks for cortical and subcortical grey and adjacent white matter using SPM neuromorphometrics atlas for tissue‐specific analysis of MT parameters (see supplementary methods in Data S1). Finally, normalised MT maps were smoothed using tissue‐weighted smoothing (7 mm FWHM) to preserve MT values in GM and WM, respectively (Draganski et al., 2011; Tabelow et al., 2019).

### 2.4 Longitudinal MT image analyses

In order to quantify myelin development, we took advantage of the observational accelerated longitudinal design of the study. We focused on how the brain changed over study time (study visit) and with age of the participants (Ziegler et al., 2019). Here we aimed to establish associations of childhood‐SED with MT differences and its changes over study visits. To do so, we used SPM's efficient Sandwich Estimator (SwE) toolbox for voxel‐based longitudinal image analysis (Guillaume et al., 2014, http://www.nisox.org/Software/SwE/). This so‐called marginal model describes expected variability as a function of predictors in a design matrix (such as visit, age, or SED), while additionally accounting for correlations due to repeated measurements and unexplained variations across individuals as an enriched error term (see supplementary methods in Data S1 for details).

In our analyses, we focused on factors time/visits and mean age of the individual (over all visits) on MT across the whole brain. To investigate how exposure to poverty was related to brain trajectories and altered MT changes, we enriched the models by adding a main effect (SED as measured by the NPI, as a predictor of mid‐point MT). The former indicates whether SED relates to overall MT differences across individuals accounting for other covariates, such as visit, mean age, and sex.

Moreover, we included an interaction term of SED with individual MT change over study visits (within‐subject study time). This metric allowed us to assess how myelin growth is associated with SED (e.g., lower myelin growth upon exposure to high SED). A priori, we hypothesised reduced levels of myelin and impaired myelin growth with higher SED. The effects of visit, age, sex, and nonlinearities of age‐related trajectories (e.g., in terms of age by age and age by study time interactions), and for first order interactions among all demographic variables are presented elsewhere (Ziegler et al., 2019). All analyses were carried out with scanning site, total intracranial volume and motion regressors as confounds. More mathematical details on SwE and longitudinal design specification can be found in Data S1.

We tested then whether the observed associations of SED might be explained (or potentially mediated) by further covariates, by including the latter on the same footing as SED in analyses. This tests whether observed SED‐brain associations were independent of particular covariates or alternatively both aspects might share some variance with an external predictor (such as family factors). The latter is a necessary (but not sufficient) condition for more complex models of mediation (Zhao, Lynch Jr, & Chen, 2010). All models were tested for indications of effects of sex, IQ, parental education, parental occupation, and self‐reported race. We further conducted moderation analysis in terms of indications whether above family or individual factors show a significant interaction with SED either on mean level MT or MT growth over visits. Thus, two‐way interaction with SED (e.g., parenting quality by SED) and three‐way interaction terms (e.g., parenting quality by SED by time/visit) were included in addition to all main effects, time, age, sex, and their interactions in SwE models of local MT. Finally, potentially confounding effects of alcohol consumption, ethnicity and body mass index (BMI) (Kiddle et al., 2017) were assessed by including those metrics as additional covariates in all SED related models presented in the main results of this work. We controlled for the false discovery rate (FDR, *p* < .05) during corrections for multiple comparisons in all image analyses. All analyses were conducted in voxel space and resulting statistical parametric maps (SPM) were projected then onto a surface of the template for illustration purposes alone.

### 2.5 Linear mixed effects modelling of global MT and BMI

To assess the effects of SED on global MT and on BMI, we used linear mixed‐effects modelling (LME, MATLAB R2016B's function fitlmematrix, see Data S1 for details) (MATLAB, 2018). We specified corresponding fixed effects design matrices including time, age, sex, SED, and first order interactions while accounting for confounds. Random‐effect intercepts were included and proved optimally suited using likelihood ratio tests. *T*‐values of fixed effects coefficients and corresponding (one‐sided) *p* values were calculated to test for detrimental main effects of SED and time/visit or age interactions. More information on LME and longitudinal design specification can be found in Data S1.

### 2.6 Macrostructural measures

Finally, to complement the main focus of this study in assessing SED‐related correlates of novel, quantitative, myelin‐sensitive MT (using VBQ), we also tested for previously reported relationships of SED with established metrics, that is, Voxel‐Based and global Surface‐based Morphometry (VBM & SBM). For this purpose, we used nonlinear registration to obtain normalised (grey and white matter) tissue segment maps using both within‐ and between‐subjects modulation. This was followed by Gaussian smoothing (6 mm). Moreover, cortical surface reconstructions of all participants scans were obtained (using the CAT Toolbox; for algorithm see Dahnke, Yotter, and Gaser (2013), for validation see for example, Seiger, Ganger, Kranz, Hahn, and Lanzenberger (2018)), and we estimated mean cortical thickness (averaged across the whole cortical mantle) and global cortical surface area.

## 3 RESULTS

### 3.1 Early disadvantage is associated with slower myelin growth

We obtained 497 repeated structural MRI scans on 288 (51.7% female) healthy participants between 14 and 25 years of age (see Section 2). We carried out an observational, accelerated‐longitudinal study of these community dwelling English young people, focusing on longitudinal findings. Our 'sandwich estimator' based analysis (Guillaume et al., 2014) allowed us to describe longitudinal results in terms of MT growth rate (MT_rate_, over follow‐up visits), adjusting for the participants' baseline age and taking into account curvilinear trajectories.

Our primary measure of SED, the neighbourhood poverty index (NPI) was available for all 288 participants at the time of scanning, and for 185 (45.7% female) of these participants before 12 years of age [UK Office of National Statistics (Fry, 2010); also Figure S2]. As the sample was epidemiologically representative, SED correlated with non‐white self‐declared race (for details see Figure S2c). In our scanned sample, SED was not related to IQ (*p* for the Pearson corr. between neighbourhood disadvantage before age 12 and total WASI IQ at baseline = 0.53). We examined putative explanatory variables both by entering them as covariates and moderating factors in the imaging analyses (see Section 2).

**FIGURE 1:**
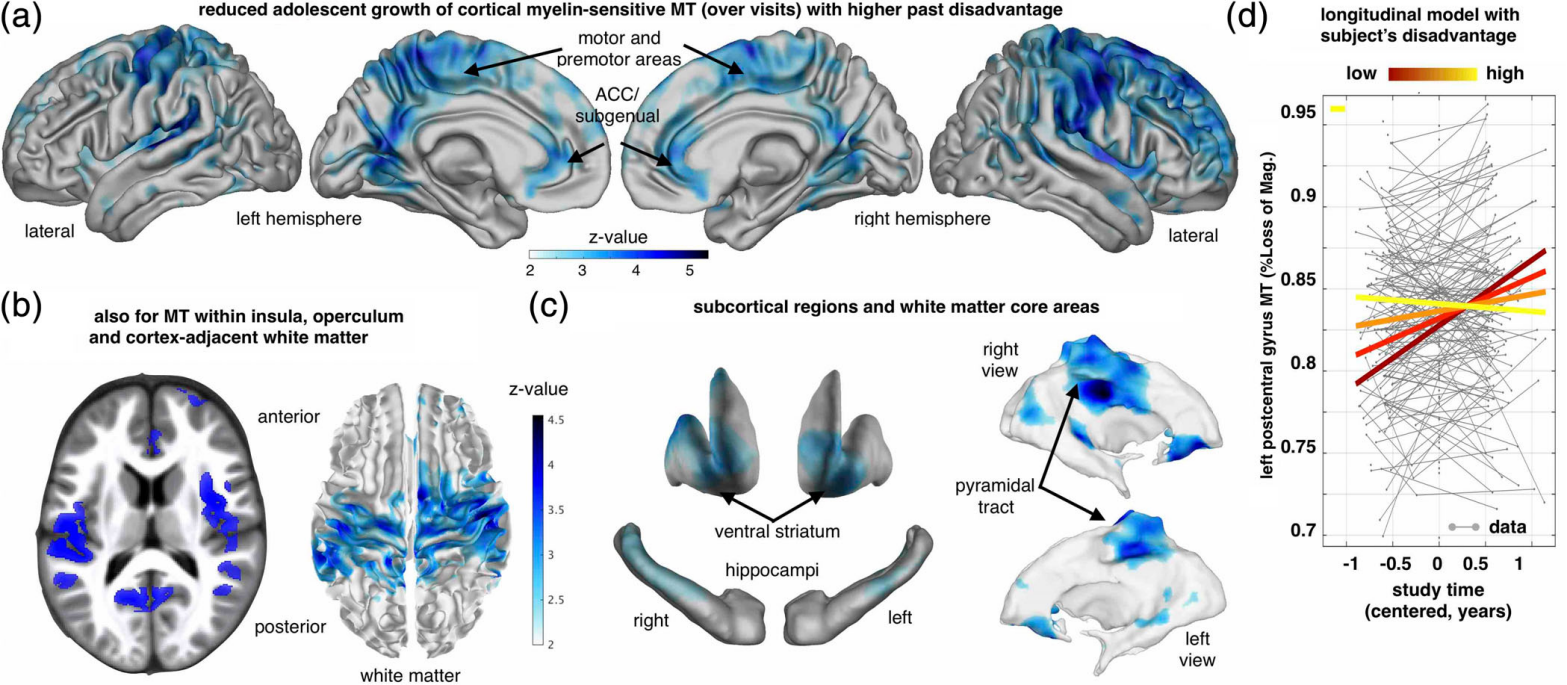
Socio‐economic disadvantage (SED) during childhood is associated with slower myelin‐related MT growth during coming of age. (a) Age‐typical growth slows down with worsening early life SED in cortex, especially in bilateral precuneus/posterior cingulate, sensory‐motor, premotor, sub‐genual, and prefrontal areas (Z‐maps showing negative SED by time/visit interactions, *p* < .05 FDR corrected, one‐sided Wald tests, accounting for age, visit/time, sex, interactions and confounds, *N* = 328/185 scans/subjects in (a)–(c), 45.7% female, see also Table S1). (b) MT increase is also reduced in insula, operculum (left panel) and the white matter adjacent to the affected cortex (right, see also Table S2). (c) Hippocampal and striatal grey matter, and core white matter regions also showed reduced increase of MT. (d). SED‐dependent rate of change of MT over visits in a region‐of‐interest sphere encompassing the central operculum/posterior insula (radius 6 mm, centre at MNI [−52, −17, 12] mm, beta ± 95%CI = −0.0089 ± 0.004). Plot shows subjects with higher SED (light yellow) compared to low SED subjects (dark red) express significantly less MT increase over visits (coloured lines in right panel indicate the interaction effect; *y*‐axis, MT; *x*‐axis, time of scan in years relative to each subject's mean age over visits). ACC, anterior cingulate cortex

Strikingly, we found that living in a more deprived neighbourhood *before* age 12 was robustly linked with reduced MT_rate_ in multiple brain areas (Figure 1 and Table S1). This supports our hypothesis that SED is associated with reduced myelin growth during late adolescent development, accounting for both baseline age and follow‐up interval (Howell et al., 2013; Jensen et al., 2017). Moreover, a reduction in MT_rate_ was observed globally, within whole‐brain grey matter (*t*[321] = −2.87, *p* = .022, one‐sided; see Section 2). All this contrasted with the impact of *current* neighbourhood disadvantage (current SED), which predicted reduced growth poorly. Furthermore, there were no brain areas where SED was significantly associated with increased MT_rate_ (or the mean MT over the study period, MT_mean_), as might be expected if some changes reflected adaptive or compensatory early growth (Ono et al., 2008; Ziegler et al., 2019).

The regions wherein early life SED correlated with reduced intra‐cortical MT_rate_, included mid‐ and posterior cingulate, precuneus, operculum, insula (Figure 1a,b) and prefrontal grey matter. Interestingly, MT_rate_ of juxta‐cortical white matter and several subcortical grey matter regions, was similarly reduced (Figure 1b,c, Table S2). These rate reductions were most pronounced in highly myelinated areas (e.g., M1, S1; for maps see for example, Glasser et al., 2014) and in regions where we previously reported a significant normative increase of MT with development (Ziegler et al., 2019).

Our hypothesis that mean level of myelin, reflected in MT_mean_ over assessment points, would show a similar reduction with SED was supported also at the global brain level, accounting for age, follow‐up visit, sex, and confounds (see Section 2). Specifically, greater SED was associated with reduced MT_mean_ (*t* = −2.15, *p* = .016, one‐sided, *df* = 321 for global grey matter; see also Table S6). However, local analyses showed no significant associations between early life SED and MT_mean_, when applying stringent FDR correction. In light of the significant global result, greater statistical power may be needed to map widespread but more subtle MT_mean_ effects that did not survive FDR correction here.

### 3.2 The contribution of family factors

Early life SED influences on brain myelination are likely to involve complex family‐related factors, indexed both by demographic characteristics but also by the quality of parental care (Whittle et al., 2017). Consequently, we examined whether parental education, parental occupation (a proxy for family income), and self‐report measures of parenting quality accounted for the effect of SED on MT increases (Ronfani et al., 2015; Sarsour et al., 2011). Parental education scores partially accounted for our MT findings, consistent with recent reports (Noble et al., 2015). While peak clusters showing an effect of SED remained significant, their extent was much reduced, especially over the medial parts of the brain. For example, the left subgenual, right medial motor and right posterior cingulate clusters were largely abolished (compare Figure 1a or Figure S4b–d with Figure S4a). Important influences over and above those indexed by parental education are likely to contribute to the effects of SED, as peak clusters remain significant upon controlling for parental education. Against our hypothesis, controlling for poorly paid parental occupation (HESA, 2003) itself had little impact on the relationship between SED and MT (Figure S4b).

**FIGURE 2:**
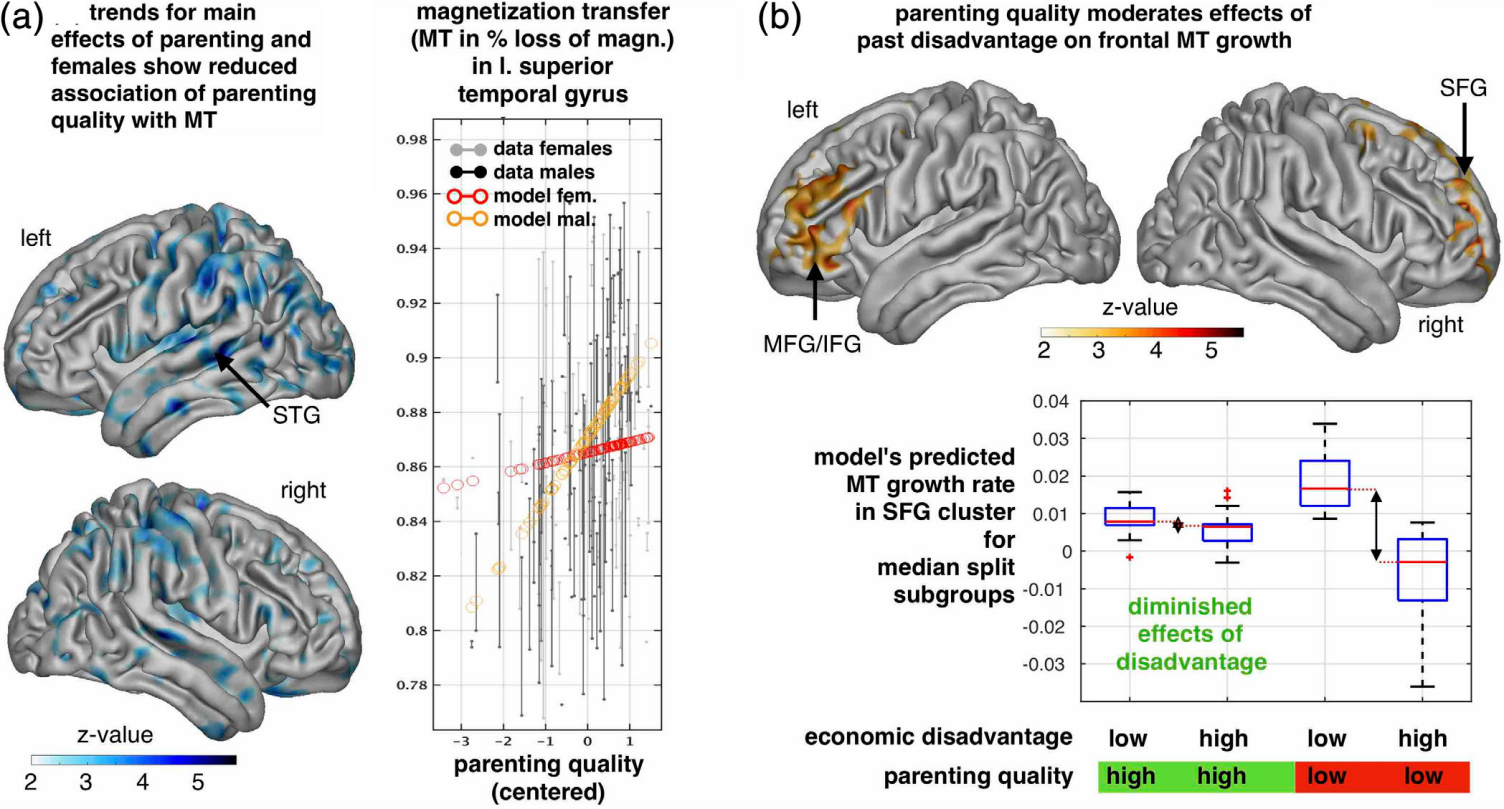
The effect of parenting quality on cortical myelin‐sensitive MT and its interaction with SED. (a) Positive parenting moderated(reduced) the detrimental effect of socio‐economic deprivation on prefrontal MT growth (Z‐maps show a positive parenting quality by SED by time/visit interaction, *p* < .05 FDR corrected, one‐sided Wald test, see also Table S3). (b) Illustration of the moderation effect (a) within right superior frontal gyrus (sphere 6 mm around MNI [27, 51, 35] mm) with SED by time and parenting by SED by time/visit interaction, beta ± 95%CI = 0.0079 ± 0.0044). We show growth rates (MTr) as predicted by the longitudinal model within median split groups of high vs. low SED and high vs. low parenting quality. Early life SED effects on MTr (illustrated by arrow) are less pronounced in family contexts with high parenting quality. (c). The effect of parenting quality on MT was significantly steeper in males compared to females (see also Table S4, Z‐maps show negative sex by parenting quality interactions, *p* < .05 FDR corrected, one‐sided Wald tests, *N* = 328/185 scans/subjects in (a)–(C), 45.7% female, accounting for age, visit/time, sex, interactions, and confounds. The right panel plots MT in orbitofrontal gyrus (6 mm sphere around peak voxel MNI [−19, 24, −20] mm, beta±95%CI = −0.0199 ± 0.0145) over parenting quality (*x*‐axis, *z*‐scored) and with adjusted data (grey/black) and model predictions (red/orange, effects of interest: intercept, parenting, sex by parenting). Higher parenting quality only showed a trend towards a positive main effect on cortical MT (*p* < .001, unc., not shown). SFG, superior frontal gyrus; MFG, middle frontal gyrus; IFG, inferior frontal gyrus; STG, superior temporal gyrus; OFC, orbitofrontal gyrus

We next examined a potential mediating role of parental caregiving quality, using our measure of overall parenting quality as experienced by the participants (see Section 2). We found that parenting quality was not associated with MT_rate_ and thus did not mediate the effect of SED on MT_rate_, although we found evidence that greater parenting quality was associated to a steeper MT increase in males (Figure 2c).

By contrast to this absence of mediation effects, we found a significant moderating effect of parenting. In other words, better parenting significantly reduced the detrimental effect of early life SED on adolescent MT_rate_. Topographically, this moderating effect was largely confined to lateral prefrontal cortical MT (Figure 2b) and subcortical MT (Table S3). Thus, SED and parental education index overlapping psychobiological influences, while better parenting quality seemingly indexes a separate influence whose presence appears to exert a protective effect in more adverse environments.

**FIGURE 3:**
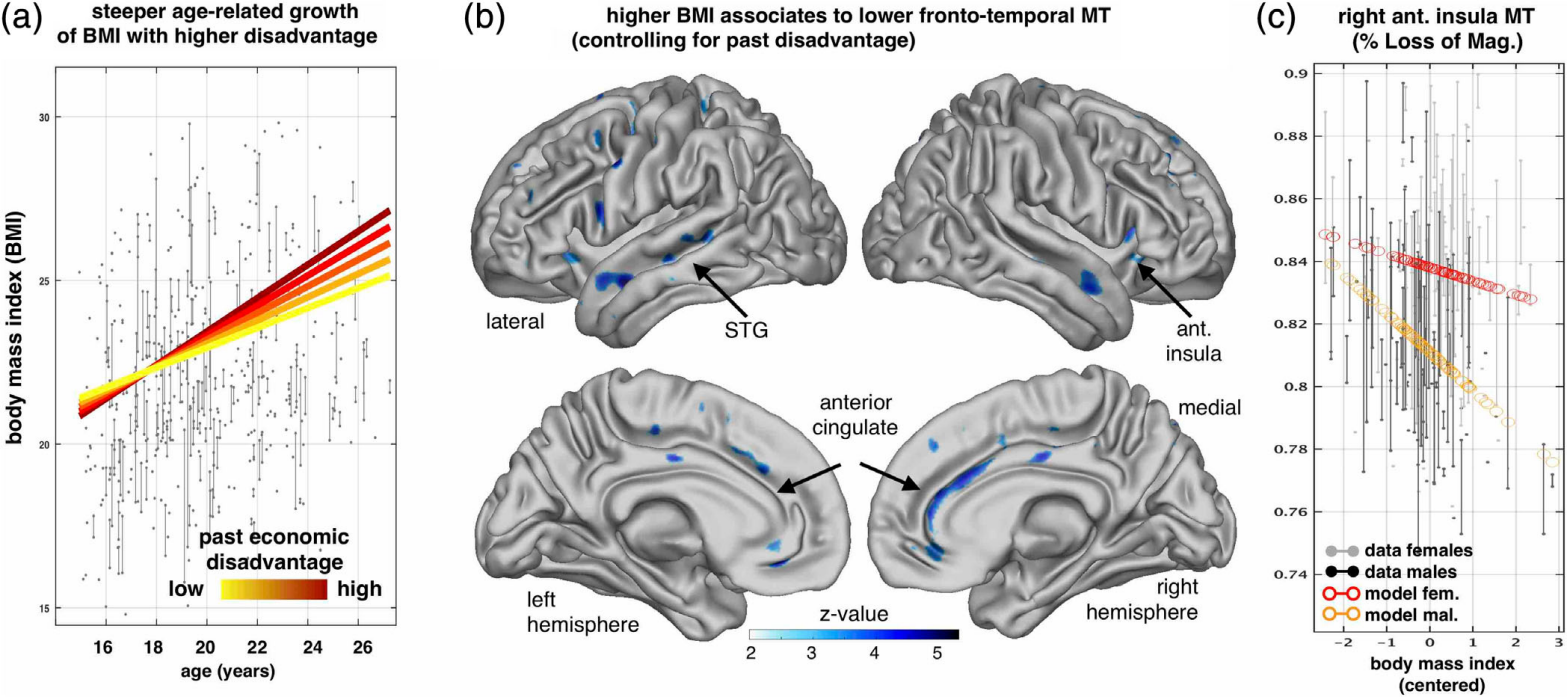
The effect of body mass index on myelin growth. (a) Early life SED is associated with faster gain of body mass index (BMI) during youth. Linear‐mixed modelling revealed positive age effects on BMI (beta ± SE = 0.021 ± 0.009, *t* = 4.6, *p* = 3.9e − 6, two‐tailed, *df* = 559), positive main effects of early life SED on BMI (beta ± SE = 0.049 ± 0.024, *t* = 2.05, *p* = .0407, two‐tailed, *df* = 559, *N* = 568/384 observations/subjects) and a steeper age‐related increase with higher SED (*t* = 2.2, *p* = .0265, *df* = 559, two‐tailed), accounting for age, visits, sex, and interactions. (b) Greater BMI is associated with lower cortical myelin‐sensitive MT in the anterior cingulate, superior temporal, anterior insula cortex (Z‐maps testing for negative BMI effects, *p* < .05 FDR corrected, one‐sided Wald tests, *N* = 277/155 scans/subjects, 47.5% female, accounting for age, visit/time, sex, early life SED, interactions and confounds). (c) Right panel shows the plot of MT in insula (6 mm sphere around peak voxel MNI [29, 23, 13] mm, beta ± 95%CI = −0.009 ± 0.0043) over BMI (x‐axis, centred) and with adjusted data (grey/black) and model predictions for sexes (red/orange, effects of interest: intercept, BMI, sex by BMI). The decline of MT with higher BMI is steeper in males than females. See also Table S5. STG, superior temporal gyrus

We also tested whether the association of MT trajectories with early life SED would be partially accounted by differences in alcohol consumption, ethnicity and body mass index (BMI) (Figure S5). BMI might be particularly relevant for myelination as it has been previously associated with deviant white matter development (Kullmann et al., 2015). Developmentally, BMI increased with age during adolescence. However, it increased faster the greater the degree of early SED (Figure 3). Correcting for age and variables of no interest (see Section 2), greater BMI was associated with lower MTm in anterior insula, anterior cingulate and other areas (Figure 3b,c). However, BMI did not account for the relationship between SED and MT_rate_.

## 4 DISCUSSION

We show that early socio‐economic disadvantage is associated with altered trajectories of neurodevelopment (Johnson et al., 2016). Early disadvantage correlated with reduced global grey matter myelin and, most importantly, with reductions in myelin growth longitudinally, as indexed by the sensitive marker magnetization transfer saturation, MT. Reminiscent of other developmental findings (Sheikh et al., 2014; Whittle et al., 2017), parental education, but not other predictors, accounted for much of this effect. Better parenting moderated the relationship, lessening the effect of economic disadvantage. As expected, increased BMI also predicted variation in myelin development, but the effect of BMI on myelination was independent of the effect of SED.

We observed widespread reductions in a measure of myelin growth, specifically MT rate of increase, with development. These effects involved grey matter of medial and lateral somatosensory and motor areas, precuneus, operculum, posterior and middle cingulum, insula and prefrontal cortex (Figure 1). They also involved white matter deep to motor / somatosensory areas and the pyramidal tract. In subcortical grey matter, posterior hippocampi and ventral striatum were similarly affected. Thus, the effects of SED on myelin growth are widespread but spatially organised in a pattern that requires further understanding.

As our sample was selected to be healthy, slower myelination might constitute neurodiversity rather than neuropathology. Speculatively, slower growth in sensory‐motor areas might represent a levelling‐off after faster, earlier growth (Ziegler et al., 2019), possibly accompanying skilled motor endeavours in economically disadvantaged young people. Diversity can be seen as biologically encoding predispositions about optimal choices given one's context, technically prior beliefs (Moutoussis et al., 2018). Such predispositions may confer adaptation, maladaptation (Jensen et al., 2017) or both, depending on how applicable they are to the current environmental context of a young person. For example, the presumption “a bird in hand is better than two in the bush” (cf. temporal discounting) may be adaptive, but only if one cannot learn to net both birds in the bush.

Pragmatically, the fact that we observed only reductions in MT_rate_, that these were extensive, and that they related to early but not current SED, suggests that the observed changes are maladaptive. Here research should test specific functional hypotheses about slower myelination. The posterior cingulate is an interesting example. Functions associated with this area include metacognition and self‐awareness, so that slower myelination might contribute to some of the complex influences of SED on person‐centred cognition that have been reported (Sheehy‐Skeffington & Rea, 2017). Generally, it will be important to establish how SED dependent myelination longitudinally impacts on IQ, on specific sensory‐motor, emotional and cognitive functions.

Turning to the pathways linking SED to slower myelination, these may possibly involve early developmental stressors which are more common among disadvantaged children. Relatedly, primate studies show that early stressors (Howell et al., 2013) have an impact on brain myelination. Intriguingly, neighbour‐level disadvantage was a more powerful predictor of slowed MT increase than parental occupation (Figure S4). To the extent that current parental occupation is an indicator of family resources available during childhood, this is rather surprising. However, resource availability may not be the dominant factor in populations without high levels of deprivation (Figure S2). In primate variable foraging demand (VFD) experiments, it is resource unpredictability (Coplan et al., 2006), rather than average resource availability, that affects infant neurodevelopment. This might suggest that neighbourhood SED exerts stress by exemplifying economic unpredictability for all, not just the poorly paid.

It might be hypothesised that IQ would reflect myelination patterns associated with disadvantage, as other brain structural measures have been found to mediate a relationship between socio‐economic status and IQ (McDermott et al., 2019). In our study, however, IQ scores did not account for the effect of SED. Notably, previous research often directly incorporated parental education as a measure of SED, which is likely to strengthen the apparent relation between IQ and SED. IQ and socio‐economic status have been claimed to share similar genetic determinants (Trzaskowski et al., 2014). An argument here might be that genes directly contributing to parental socio‐economic success might also contribute to differences in brain structure, as indexed by IQ. In other words, SED and brain growth might be associated through horizontal genetic pleiotropy (Figure S8). Our results suggest that such a causal argument is likely to be much too simplistic, as genes directly contributing to both (neighbourhood) SED and MT growth would then underpin our IQ measures. Genetic, and indeed environmental, antecedents of myelination may give rise to an intermediate phenotype, on which SED influences operate to affect neurodevelopment (Figure S8). In this case SED would disproportionally affect the genetically vulnerable (Gage, Smith, & Munafo, 2016).

Parental education was a key predictor and accounted substantially for the effects of SED (Figure S4a), suggesting these two processes index overlapping biological pathways, reducing MT_rate_. The shared effect of parental education was unlikely to be due to intelligence or other individual‐level factors that we examined, as these were independent of the effect of SED (Figure S5). Given the substantial animal and human imaging literature (Jackowski et al., 2011; Whittle et al., 2017), we turned to the quality of parenting to understand the effect of SED. Against our hypothesis, self‐reported parenting behaviour did not account for the association between SE and MT_rate_. However, we did find evidence that parenting moderated the association between SED and MT_rate_, consistent with other studies (Luby et al., 2013; Whittle et al., 2017). These findings are important, although we note that the topographical distribution of this moderating effect had modest overlap with the regions identified in our analysis of the SED‐MT_rate_ association, being confined to lateral prefrontal cortices.

How parental education partially explains the effect of SED is likely to include diverse factors not captured in our measures of parenting quality. Interestingly, the lack of explanatory power of parental occupation provides preliminary evidence against a simple effect of overall material provision or status, replicating the work of Whittle and co‐workers (Whittle et al., 2017). In the context of material provision, primate VFD studies suggest that the negative impact of “variable foraging demand” is mediated by maternal preoccupation induced by insecurity (Coplan et al., 2006), consistent with positive parenting modestly mitigating the effects of SED (Figure 2). However, animal studies use focused, high‐impact stressors, unlike the complex multitude of factors involved in SED, especially in a healthy sample like ours where severe adversity was under‐represented (Figure S2). Therefore, future research should test the specific causal role of socioeconomic insecurity on brain development versus other candidate factors such as the provision of enriched early home and school environments, factors such as family chaos (Doom et al., 2018) and parental conflict, or factors associated with early peer relationships.

We did not replicate reports connecting SED to macroscopic measures such as grey matter volume or surface area. Our smaller sample, though a limitation, is likely to mean that myelination effects are statistically stronger than macroscopic effects. Our results are also consistent with myelination being a more specific or developmentally more sensitive process than macroscopic measures (Grydeland, Walhovd, Tamnes, Westlye, & Fjell, 2013; Ziegler et al., 2019).

One limitation of our study, which can be addressed in future research, is that early SED was assessed retrospectively. Additionally, although chronological age is important, future studies would benefit from use of endocrinologically precise pubertal stage as a developmental index. Research would also benefit from including more deprived populations, such as samples from low‐income countries and cohorts affected by serious economic recessions before the age of 12. Our findings can usefully inform future research regarding public health. They indicate that intervention studies aiming to reduce SED and/or improving parental education and parenting could provide a context to prospectively examine an impact on myelination.

## 5 CONCLUSIONS

In this study, we show that neighbourhood deprivation during development is associated with a slower myelin growth during adolescence and young adulthood. This effect was independent of baseline IQ, race or parental occupation. Parental education statistically explained much of the effect and offers clues about potential causal mechanisms. Causation, functional consequences and policy implications of these findings provide a fertile context for future investigations.

## CONFLICT OF INTERESTS

The authors have no financial or non‐financial competing interests to declare.

## CODE AVAILABILITY

Custom made SPM pipeline code for longitudinal VBM and VBQ processing is provided along with the manuscript (https://github.com/gabrielziegler/gz/tree/master/nspn_mpm_prepro_code_and_example). The code aims at transparency of applied procedures but is not intended for clinical use. It is free but copyright software, distributed under the terms of the GNU General Public Licence as published by the Free Software Foundation (either version 2, or at your option, any later version). For any questions and requests please contact gabriel.ziegler@dzne.de.

## Supporting information


**Data S1** Supporting Information.

## Data Availability

Whole‐brain results are available for inspection online on Neurovault (https://neurovault.org/collections/JYPTMYJO/). Our participants did not give informed consent for their measures to be made publicly available, and it is possible that they could be identified from this data set. Access to the data supporting the analyses presented in this paper will be made available to researchers with a reasonable request to openNSPN@medschl.cam.ac.uk or the corresponding / first authors [M.M., G.Z.].
