# Supplementary material for: Childhood socio‐economic disadvantage predicts reduced myelin growth across adolescence and young adulthood
Source: Hum Brain Mapp. 2020 May 20;41(12):3392–402. doi: 10.1002/hbm.25024 (PMC7375075; doi:10.1002/hbm.25024)
Supplement: Supplementary file 1 — Data S1 Supporting Information. [file HBM-41-3392-s001.docx]

### A. Supplement to Materials: participant sample

| 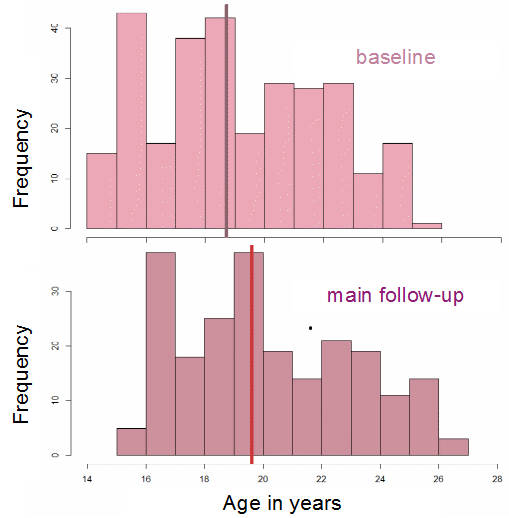 |
| --- |
| **Figure S1.** **Age distribution of participant sample.** Top – baseline scan; bottom; main follow-up scan. Vertical lines: median ages at each scan. The distributions are similar, but the median is shifted to 14.5 months older (min follow up interval =11.6 months, max=28.2 months). In addition, scans of satisfactory quality were obtained at a small subsample of 21 participants at a short, approximately 6-month follow up. They had a median age of 19.1 years, range 15.2-23.5, interquartile 16.9-21.3 years. |

| 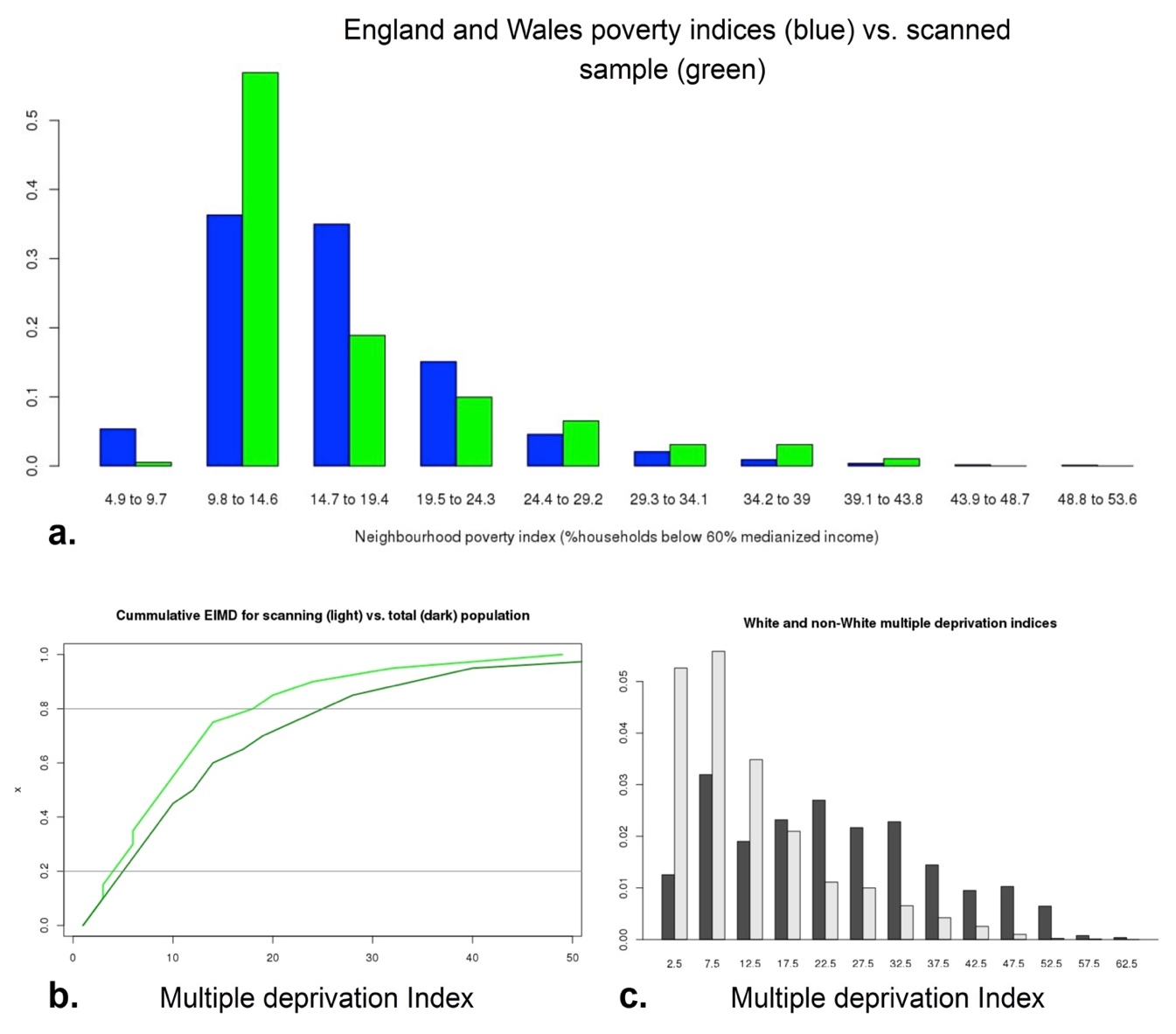 |
| --- |
| **Figure S2.** **The scanned sample was approximately representative of the English population with respect to socio-economic deprivation.** **a.** Our study sample (in green) contained fewer economically disadvantaged participants than England and Wales as a whole (blue) **b.** Cumulative distribution of SED based on the 2015 English Index of Multiple Deprivation measure (EIMD; provided by the Office of National Statistics, UK) related to the neighbourhood poverty index (Pearson *r*=0.77, p<1e-10). We use EIMD in this panel as it was available for the entire N=2400 ‘community sample’ from which the scanned sample was recruited. Again, the scanned sample (light green) had a higher density of low disadvantage and lower levels of median EIMD, Wilcoxon p=1.7e-7. In order to obtain an index of psychometric differences, we also estimated the mean mood score (Mood and Feelings Questionnaire score, MFQ). The mean MFQ of the scanned sample was also better than that of the non-scanned, but the expected relationship between increasing SED and worse mood was similar in the scanned and non-scanned samples (Pearson correlation 0.15 and 0.09, uncorr. *p* 0.01 and 0.0003 respectively). Similarly, the relationship between IQ and SED was similar, and significant, in the scanned sample compared to a non-scanned , N=486 sub-sample of the non-scanned group (but still volunteering to attend the laboratory; Pearson r = -0.13 for both, p=0.0012 uncorr. for these two groups together). **c.** The role of race/ethnicity. Those identifying as 'white British' (light grey) were economically advantaged compared to non-whites (dark bars). In the community sample, non-whites also had greatly increased SED compared to whites (EIMD: Wilcoxon test p < 1e-15). |

***Socioeconomic Indices***

The key measure used was the neighbourhood poverty index (NIP), provided by the British Office of National statistics ONS. This was the proportion of households within a fixed geographical area to which the participant’s post-code belonged. The geographical area is termed a 'Middle Layer Super Output Area’ (MSOA). England and Wales were divided into MSOAs in the aftermath of the 2001 census to track socioeconomic tends. MSOAs were composed of smaller units (so each post-code belonged to just one) and were geographically contiguous, but their boundaries were then traced so that they would be (i) socially relatively homogeneous and (ii) would have a minimum population of 5000 (mean 7200). The NIP provided at the time of the study by the ONS was the proportion of households in the MSOA below 60% of the median for England and Wales. The income in questions was the ‘net equivalised household income after housing costs’. That is, housing costs were themselves detracted from income and then the number of people in the household was taken into account. For example, the poverty threshold for a household of two people without depedants in 2008 was GBP 199 per week (Fry, 2010).

We also used the parents’ occupational class (SOC2000; HESA, 2003), and more specifically the ‘Major group classification’ of SOC2000, as a proxy of economic disadvantage and approximate measure of household income. This major group classification is: 1 Managers and Senior Officials; 2 Professional Occupations; 3 Associate Professional and Technical Occupations; 4 Administrative and Secretarial Occupations; 5 Skilled Trades Occupations; 6 Personal Service Occupations; 7 Sales and Customer Service Occupations 8 Process, Plant and Machine Operatives; 9 Elementary Occupations. Parents (or participants, if over 18) provided a specific occupation, e.g. ‘lawyer’, which was mapped for the scanning sample to the numerical codes above by hand, according to the detailed instructions of HESA ( [www.hesa.ac.uk/support/documentation/occupational/soc2000](http://www.hesa.ac.uk/support/documentation/occupational/soc2000) ).

***Power considerations & analysis***

With respect to power analysis, there was no previous data pertaining to MT saturation in adolescents to provide a rigorous basis for sample size estimation. However, a roughly comparable older study achieved considerable precision using a cohort of 387 subjects from which 829 MRI scans were acquired (Giedd, 2004). We thus aimed to recruit the maximum of 300 healthy participants that was logistically feasible within our time and resource constraints, and to optimise power by selecting age and follow up study parameters as follows. Previous data was obtained (Giedd, 2004) and different study designs were simulated based on cross-sectional N=300 and using global grey matter volume as a measure to which quadratic growth functions of age were fitted. The study design was then optimised with respect to the minimum and the width of recruitment age bins, number and interval between follow-ups, to minimize the variance of the quadratic growth parameters. Simulations showed that no substantial gains in efficiency (or indeed losses) would be made by reducing overall age range below 10 years, or the age-bin width below 2 years, or increasing the number of follow-up waves above 2. Parameter accuracy improved with baseline to follow-up interval, deteriorating substantially below an interval of 6 months. Therefore, we recruited 300 participants in 5 equally spaced two-year bins, matched for sex and aimed for an inter-scan interval of 12-18 months.

### B. Supplement to Methods: Statistical modelling

***MPM Imaging protocol***

Three multi-echo FLASH scans were acquired with T1-, PD-, and MT-weighting by appropriate choice of the repetition time (TR) and the flip angle α: TR/α = 18.7 ms/20^◦^ for the T1w scan and 23.7 ms/6^◦^ for the PDw and the MTw scans. The MT-weighting was achieved by applying an off-resonance Gaussian-shaped RF pulse (4ms duration, 220^◦^ nominal flip angle, 2 kHz frequency offset from water resonance) prior to the excitation. Multiple gradient echoes were acquired with alternating readout polarity at six equidistant echo times (TE) between 2.2 and 14.7ms for the T1w and MTw acquisitions and at 8 equidistant TE between 2.2 ms and 19.7 ms for the PDw acquisition. Further acquisition parameters were as follows: 1 mm isotropic resolution, 176 sagittal partitions, field of view (FOV) = 256 × 240 mm, matrix = 256 × 240 ×176, parallel imaging using GRAPPA factor 2 in phase-encoding (PE) direction, 6/8 partial Fourier in partition direction, non-selective RF excitation, read- out bandwidth BW = 425 Hz/pixel, RF spoiling phase increment = 50^◦^. The total acquisition time of the above protocol was optimized to ∼19 min. More details can be found in Weiskopf et al. (2013).

***Longitudinal MRI data processing using SPM***

Since longitudinal neuroimaging is prone to artefacts due to registration inconsistency, scanner inconsistencies and age-related deformations of the brains, pipelines were carefully designed in order to detect the changes of interest and achieve unbiased results.

First, we performed symmetric diffeomorphic registration for longitudinal MRI (Ashburner and Ridgway, 2012), combining non-linear diffeomorphic and rigid-body registration and a correction for intensity inhomogeneity artefacts. The optimization is realized within one integrated generative model and provides consistent estimates of within-subject brain deformations over the study period. The registration model also creates a midpoint image for each subject and the corresponding deformation fields for every individual scan. Large sample longitudinal processing parameters (noise-model and warp regularization) were optimized for detection of significant microstructural change within cortical and subcortical gray matter and cortical white matter regions using a parameter grid search within a test-sample of 20 subjects.

Second, we applied SPM12's Computational Anatomy Toolbox (CAT, r1207, Structural Imaging Group, http://dbm.neuro.uni-jena.de/cat12/) segmentation to each subject's midpoint image, which assumes every voxel to be drawn from an unknown mixture of gray matter (GM), white matter (WM), and cerebrospinal fluid (CSF) tissue classes. Earlier results demonstrated that MT maps are highly suitable for automated segmentation in multi-subject morphometric studies, showing improved GM tissue contrast in subcortical structures (Helms et al., 2009). The applied segmentation algorithm performs a partial volume estimation (PVE) to account for mixed voxels with two tissue types (Tohka et al., 2004). Moreover, the segmentation is based on an adaptive maximum a posteriori (AMAP) approach (Rajapakse et al., 1997), and subsequent application of a hidden Markov random field model (Cuadra et al., 2005). Notably, the applied AMAP estimation does not rely on tissue priors, which overcomes potential bias due to the application of inappropriate tissue priors in adolescent maturing subjects with different to adult brain anatomy.

Third, non-linear template generation and image registration to MNI space was performed using the individual midpoint GM and WM tissue maps and diffeomorphic registration using geodesic shooting (Ashburner, 2009, 2011). Quantitative MT maps from all time-points were normalized to the MNI space using (within- and between-subjects) transformations obtained in previous steps.

Fourth, since our study aimed at local statistical analysis of voxel-based trajectories of quantitative MT parameters within GM and WM tissue classes, the normalized MT maps were smoothed using previously established (Draganski et al., 2011) tissue-weighted-smoothing with a Gaussian kernel of 6 and 7 mm full width at half maximum (FWHM) for subcortical and cortical regions, respectively.

Fifth, in order to avoid biasing results by image artefacts (e.g. due to movement, segmentation or normalization errors), the MPM maps were carefully checked manually before and after longitudinal registration by an expert [author GZ]. Additionally, the obtained normalized and smoothed MT data (in MNI space) was quality checked using statistical covariance-based sample inhomogeneity measures (as implemented in the CAT toolbox) to exclude subjects with extremal overall deviation of quantitative values due to acquisition or processing artefacts. Further quality checks were performed based on excluding 10% worst scans using a during scan motion proxy based on R2* decay model (for details see also Castella et al. (2018) and below section on modelling of covariates and confounds).

Finally, in addition to the main focus of this study, i.e. assessing changes of novel myelin-sensitive MT (using VBQ), we also performed global analysis of more conventional Voxel-based and Surface-Based Morphometry (VBM and SBM) metrics. Using default pipelines in SPM’s CAT12 toolbox we derived global grey and white matter volumes, mean cortical thickness and global surface area of each participant. CAT’s surface reconstruction algorithm has be described in detail in Dahnke et al. (2013) and successfully validated against freesurfer (Seiger et al., 2018). All processed MT saturation data and morphometric maps for subsequent longitudinal modelling steps were obtained using the above steps.

***Regions of interest (ROI)***

The primary focus of this paper is Voxel-based Quantification (VBQ) analysis of myelin-sensitive MT within the cortical and subcortical gray matter and the cortex-adjacent white matter areas. First, in order to define disjoint but adjacent gray and white matter regions for voxel-based analysis in the MNI template, the gray and white matter tissue classes of the template were thresholded with 0.5, resulting in an approximately symmetric GM/WM boundary, i.e. with probability approximately 0.5 for each tissue class for voxels on the boundary. The resulting (non-overlapping) canonical grey and white matter tissue masks are not expected to be biased towards either grey or white matter and thus avoid over- or underestimation on both tissue classes. Notably, tissue-weighted smoothing applied to MT images did preserve quantitative values on both sides of the GM/WM boundary with MT in WM being significantly higher than in GM (see Figure 2 in Ziegler et al., 2019). Second, we used the SPM neuromorphometrics atlas to separate cortical from subcortical areas with respect to gray and white matter. Analysis masks for grey and white matter were derived using morphometric operations such as dilation and erosion. E.g. dilation of GM cortical mask by (extended) by 5 voxels intersected with WM tissue class from the study wise template was used to define cortex adjacent white matter. Analyses from grey matter and white matter MT were therefore performed separately. Surface rendered versions of the study-wise voxel-based templates were used to project the statistical parametric maps for more compact wholebrain results illustration (using CAT’s vol2surf routines).

***Linear-mixed effects (LME) modelling for questionnaire scores and global brain parameters***

LME is a widely used analysis technique for univariate (scalar) or mass-univariate neuroimaging data when repeated measurements are available (Pinheiro & Bates, 2000, Bernal-Rusiel, et al., 2013). LME typically assumes modelling subject *i*’s data as *y_i_ = X_i_β + Z_i_b_i_ + ε_i_,* with fixed effects design *X_i_*, random effects design *Z_i_,* and residuals *ε_i_.* Residuals were assumed to have zero expectation and an error covariance matrix *σ^2^I*. Moreover, random effects follow *b_i_ ~ N(0,σ^2^D)* with Cholesky parametrization, *D=LL^T^* with lower triangular *L.* Throughout this paper, we used LME as implemented in MATLAB (R2016b; function ‘fitlmematrix’) using Restricted Maximum Likelihood (ReML) optimization of the above model under the full covariance. Notably, implementing linear trajectory models in this framework, allows for intercept slope correlations (d_12_=d_21_≠0). Thus, inclusion of random slopes results in two additional hyperparameters, which might result in over-parametrization for a given dataset. We therefore compared this model to one with uncorrelated random effects using simulated likelihood-tests (using MATLAB’s ‘compare’ function). Comparison of multiple models with varying numbers of random effects was performed using likelihood ratio tests (p<0.05). For inference on fixed effects about main effects, interactions, quadratic components, we used linear contrasts. LME was used for global (scalar) analysis of MT and morphometric parameters (such as global surface area, average thickness) in relation to SED.

***Longitudinal image modelling using the Sandwich Estimator (SwE)***

The prevailing longitudinal image analysis method (in the sense of mass-univariate approaches) in the context of brain development uses LME. However, this approach often makes restrictive or unrealistic assumptions, e.g. compound symmetry (Fitzmaurice et al., 2008). Moreover, LME is based on iterative algorithms, which are not guaranteed to converge in all voxels of the search space. In this study, we therefore modelled the image data using a state-of-the-art analysis method recently introduced, the longitudinal Sandwich Estimator (SwE), <http://www.nisox.org/Software/SwE>, SPM toolbox, Guillaume et al., 2014). Using this so-called marginal model, one describes the data of individual *i* as *y_i_ = X_i_β + ε_i_^*^,* i.e. based only on a fixed effects design matrix *X_i_.* The randomness is treated as nuisance and modelled by marginal error terms *ε_i_^*^* with mean 0 and positive semi-definite covariance *V_i._* Marginal models do not require specification of random-effects and allow unbiased population-average inference and predictions about brain change in certain sub-groups or in relation to covariates.

The modelling approach first estimates the parameters of interest with a simple ordinary least squares $\hat{\beta}=\left( \sum_{i=1}^{m} X_{i}^{'}W_{i}X_{i} \right)^{-1}\sum_{i=1}^{m} X_{i}^{'}W_{i}y_{i}$ while working matrix *W_i_* is assumed to be identity in this application. Second, the approach estimates variances/covariances with the specific estimator, which accounts for the within-subject correlation existing in the longitudinal image observations. The covariance of the parameter estimate is $Cov\left( \hat{\beta} \right)=\left( \sum_{i=1}^{m} X_{i}^{'}W_{i}X_{i} \right)^{-1}\left( \sum_{i=1}^{m} X_{i}^{'}W_{i}V_{i}W_{i}X_{i} \right)\left( \sum_{i=1}^{m} X_{i}^{'}W_{i}X_{i} \right)^{-1}$ with separate estimates of subject *i*’s covariance *V_i_* accounting for the fact that we have 2 or 3 scans per person available in our application within the accelerated longitudinal design. Due to our large available sample, no small sample size corrections were necessary. The SwE method has been shown to allow unbiased estimation of within- and between-subject effects as columns of the same design matrix using longitudinal image observations. Inference on linear hypotheses $H_{0}:C\beta=0$ are based on Wald tests (for further details on inference see Guillaume et al., 2014). Because of its advantages and efficiency for the *mass-univariate* voxel-based modelling scenario, we here applied SwE instead of LME. The design specification followed similar guidelines for SwE and LME and had as follows.

***Longitudinal design specification: Separation of study time/visits and mean age***

Following common guidelines for longitudinal design specification (freesurfer: <https://surfer.nmr.mgh.harvard.edu/fswiki/LinearMixedEffectsModels>, Bernal-Rusiel et al., 2013, SPM analysis practice <http://www.fil.ion.ucl.ac.uk/spm/doc/books/hbf2/> and Guillaume et al., 2014) the longitudinal design matrix *X = [intercept, X_eoi_, X_eni_, X_conf_]* (obtained from stacked individual design matrices *X_i_*) was carefully specified to include effects of interest *X_eoi_*, covariates of no interest *X_eni_* and potentially confounding effects *X_conf_*. The particular choice of these matrices is motivated and specified as follows. The clear distinction between variables of interest and no interest is partially arbitrary and follows naming conventions, since both sets of factors are similarly included and estimated and their contributions modelled explicitly. The variables of interest are the ones close to our main hypotheses and are reported in some detail.

As outlined in detail in Guillaume et al. (2014), for a given longitudinal observational design, the participants’ covariate ‘*age at scan*’ (e.g. available at *0, 6, 12, 18* months after baseline) has two separable components, a between-subject component and a within-subject component, indistinguishable with cross-sectional samples. The between-subject component (further referred to as *age_mean*) is purely cross-sectional and can be obtained by considering only each participant’s mean age across all their acquired scans. In contrast, the within-subject component (referred to as *time/visit*) is purely longitudinal and considers the actual effects of study time/visit on each individual, and follows by subtracting the mean age of a participant from its age covariate. In this study, we consistently decomposed a centred ‘*age at scan*’ variable (subtracting its overall mean $\overline{age}$) using this idea, and the separate (within- and between-) subject components for subject *i* (*=1, ..., 288*) and visit/timepoint *j* (*=1, 2, 3*) were obtained as follows

$${age}_{ij}-\overline{age}=\left( {age}_{ij}-\overline{age}_{i} \right)+\left( \overline{age}_{i}-\overline{age} \right)={time}_{ij}+{{age}_{mean}}_{,i}$$

with centred study time/visit variable ${time}_{ij}$ and the mean age of each participant ${{age}_{mean}}_{,i}$. If we would only use the original ‘*age at scan*’ covariate in the longitudinal design matrix *X*, we would implicitly assume that the effects on the images are the same for both above components. However, the effects of within- and between subjects component can be very different as shown by Neuhaus and Kalbfleisch (1998). Cross-sectional components might be affected by cohort effects (i.e. different populations) which then would lead to biased estimates. More specifically, (1) overestimation of ageing-related effects due to e.g. cohort differences (Hoffmann et al., 2011, Sliwinski et al., 2010) (2) underestimation e.g. due to selective attrition, training effects and (3) even sign reversals of between- and within-subjects effects have been observed in previous analyses (Kievit et al., 2013). Furthermore, (4) cross-sectional data complicate addressing some of the fundamental questions in the field: How do brain changes (over time) differ across individuals- or groups, typically in relation to a third variable (Raz & Lindenberger, 2011) e.g. trait dimensions?

Thus, here we followed the recommendation of Guillaume et al. (2014) and systematically split the age covariate into its between- and within-subject components and consistently include both in the design matrix *X*. As shown by Guillaume et al., this separation improves the efficiency of the longitudinal Sandwich Estimator (SwE) modelling method (applied below) when assuming an identity working covariance matrix, showing that the SwE is nearly as efficient as Generalized Least Squares estimates. This finding also suggests the importance of centring covariates when inference is made on the intercepts, time etc. by avoiding increasing variance due to correlated regressors such as the intercept, time and other covariates of no interest. In addition to the presented voxel-based neuroimaging analysis in this paper, the outlined splitting of *age_mean* and *time/visit* was also applied in all linear-mixed effects (LME) models presented here. Details on observed effects of development (including test for non-linearities) and sex effects in the same sample were reported in Ziegler et al. (2019).

***Longitudinal image analysis: Modelling effects of interest***

The considered effects of interest of this particular study are effects of childhood and current SED and their interaction with study *time/visit*, i.e. *X_eoi_ = [SED, time/age by SED interaction].* For example, time or age interactions allow testing for different rates of change (over visits or with age) in subjects with higher or lower expression of SED. In this study, we use *time/visit* to refer to the individually-centred study *time* as introduced above (after separation from *age_mean* differences, i.e. time of scan in years relative to each subject’s mean age over all visits).

***Longitudinal image analysis: Modelling effects of covariates and confounders***

Given that quantitative MRI measures during development are expected to vary with further covariates known by design, known variability across subjects, undesired effects of intra-scan motion, scanning site and global brain variables, we explicitly model variability due to other covariates (or ‘effects of no interest’ for the main hypothesis) *X_eni_ = [time/age, sex]* as well as confounders *X_conf_ = [total intracranial volume, site1, site2, motion proxy]* using centred variables to minimize biases induced by correlated regressors.

A well-known confounder for analysis of local structural change and neuroanatomic correlates of cognitive or other traits is that results might be induced by substantial differences in brain size. We therefore accounted for variations in a subject-specific estimate of total intracranial volume *(ticv)* in all models. Although not intended per construction, quantitative MRI features might be partially influenced by normalization and local tissue morphometry as well. This resulted in including the *ticv* consistently as covariate for VBQ. Finally, unintended measurement variations due to site-specific acquisition differences were modelled using two centred indicator variables encoding each participant’s scan location (two in Cambridge, one in London).

To account for differences between scanners, we used scanner sites as an additional covariate with (Wolfson Brain Imaging Centre) WBIC as reference site (377 out of 497 scans were acquired at WBIC site). Thus, two additional offsets to describe potential mean deviations from WBIC were included if a scan took place at UCL and CBU sites. This procedure also accounts for change of scanners over visits, because these covariates were implemented in a fully time-varying design correcting the estimated offset for each scan independently.

Furthermore, we studied time-varying motion proxy as an additional regressor in our longitudinal SwE modelling of MT. We carried out additional analyses to assess the effects of motion and to control for potential influences thereof. Recent work from Castella et al. (2018) studied prospective motion correction in the context of Multi Parameter Mapping (MPMs). More specifically, during MPM generation a multi-echo model is estimated ([www.hmri.info](http://www.hmri.info/)). The standard deviation parameter of R2* residuals in white matter areas (SDR2*) has been shown to be an accurate proxy of individual’s movement during a scan (Castella: Fig. 2, 4 & 5). We therefore excluded (on top of other artefacts) those scans with the 10% highest values of SDR2* (above value of 5). A threshold of 10% was set as it revealed a steep increase in our motion proxy for subjects exceeding this threshold. We report a positive association of SDR2* in relation with MT in Ziegler et al., (2019). To control for residual effects of motion-induced variability we included SDR2* in all MT analyses as confounding variable, rendering the presented associations linearly independent of this proxy of absolute motion. Importantly, only few findings changed slightly after inclusion of motion-related effects.

**Supplementary references on Statistical modelling**

Ashburner, J. & Ridgway, G. R. Symmetric diffeomorphic modeling of longitudinal structural MRI. *Front. Neurosci.* **6**, 197 (2012).

Ashburner, J. & Friston, K. J. Computing average shaped tissue probability templates. *NeuroImage* **45**, 333–341 (2009).

Ashburner, J. & Friston, K. J. Diffeomorphic registration using geodesic shooting and Gauss–Newton optimisation. *NeuroImage* **55**, 954–967 (2011).

Draganski, B. *et al.* Regional specificity of MRI contrast parameter changes in normal ageing revealed by voxel-based quantification (VBQ). *NeuroImage* **55**, 1423–1434 (2011).

Guillaume, B. *et al.* Fast and accurate modelling of longitudinal and repeated measures neuroimaging data. *NeuroImage* **94**, 287–302 (2014).

Kievit, R. A., Frankenhuis, W. E., Waldorp, L. J. & Borsboom, D. Simpson’s paradox in psychological science: a practical guide. *Front. Psychol.* **4**, 513 (2013).

Bernal-Rusiel, J. L. *et al.* Statistical analysis of longitudinal neuroimage data with Linear Mixed Effects models. *NeuroImage* **66**, 249–260 (2013).

Castella, R. *et al.* Controlling motion artefact levels in MR images by suspending data acquisition during periods of head motion. *Magn. Reson. Med.* (2018). doi:10.1002/mrm.27214

Helms, G., Draganski, B., Frackowiak, R., Ashburner, J. & Weiskopf, N. Improved segmentation of deep brain grey matter structures using magnetization transfer (MT) parameter maps. *NeuroImage* **47**, 194–198 (2009).

Tohka, J., Zijdenbos, A. & Evans, A. Fast and robust parameter estimation for statistical partial volume models in brain MRI. *NeuroImage* **23**, 84–97 (2004).

Rajapakse, J. C., Giedd, J. N. & Rapoport, J. L. Statistical approach to segmentation of single-channel cerebral MR images. *IEEE Trans. Med. Imaging* **16**, 176–186 (1997).

Cuadra, M. B., Cammoun, L., Butz, T., Cuisenaire, O. & Thiran, J.-P. Comparison and validation of tissue modelization and statistical classification methods in T1-weighted MR brain images. *IEEE Trans. Med. Imaging* **24**, 1548–1565 (2005).

Pinheiro, J. & Bates, D. *Mixed-Effects Models in S and S-PLUS*. (Springer Science & Business Media, 2000).

Neuhaus, J. M. & Kalbfleisch, J. D. Between- and Within-Cluster Covariate Effects in the Analysis of Clustered Data. *Biometrics* **54**, 638–645 (1998).

Hoffman, L., Hofer, S. M. & Sliwinski, M. J. On the confounds among retest gains and age-cohort differences in the estimation of within-person change in longitudinal studies: a simulation study. *Psychol. Aging* **26**, 778–791 (2011).

Sliwinski, M., Hoffman, L. & Hofer, S. M. Evaluating Convergence of Within-Person Change and Between-Person Age Differences in Age-Heterogeneous Longitudinal Studies. *Res. Hum. Dev.* **7**, 45–60 (2010).

Raz, N. & Lindenberger, U. Only time will tell: cross-sectional studies offer no solution to the age-brain-cognition triangle: comment on Salthouse (2011). *Psychol. Bull.* **137**, 790–795 (2011).

Fitzmaurice, G., Davidian, M., Verbeke, G. & Molenberghs, G. *Longitudinal Data Analysis*. (Chapman and Hall/CRC, 2008).

Dahnke, R., Yotter, R. A., & Gaser, C. (2013). Cortical thickness and central surface estimation. *NeuroImage*, *65*, 336–348. http://doi.org/10.1016/j.neuroimage.2012.09.050

Seiger, R., Ganger, S., Kranz, G. S., Hahn, A., & Lanzenberger, R. (2018). Cortical Thickness Estimations of FreeSurfer and the CAT12 Toolbox in Patients with Alzheimer's Disease and Healthy Controls. *Journal of Neuroimaging : Official Journal of the American Society of Neuroimaging*, *28*(5), 515–523. http://doi.org/10.1111/jon.12521

### C. Control analyses of MT change

| 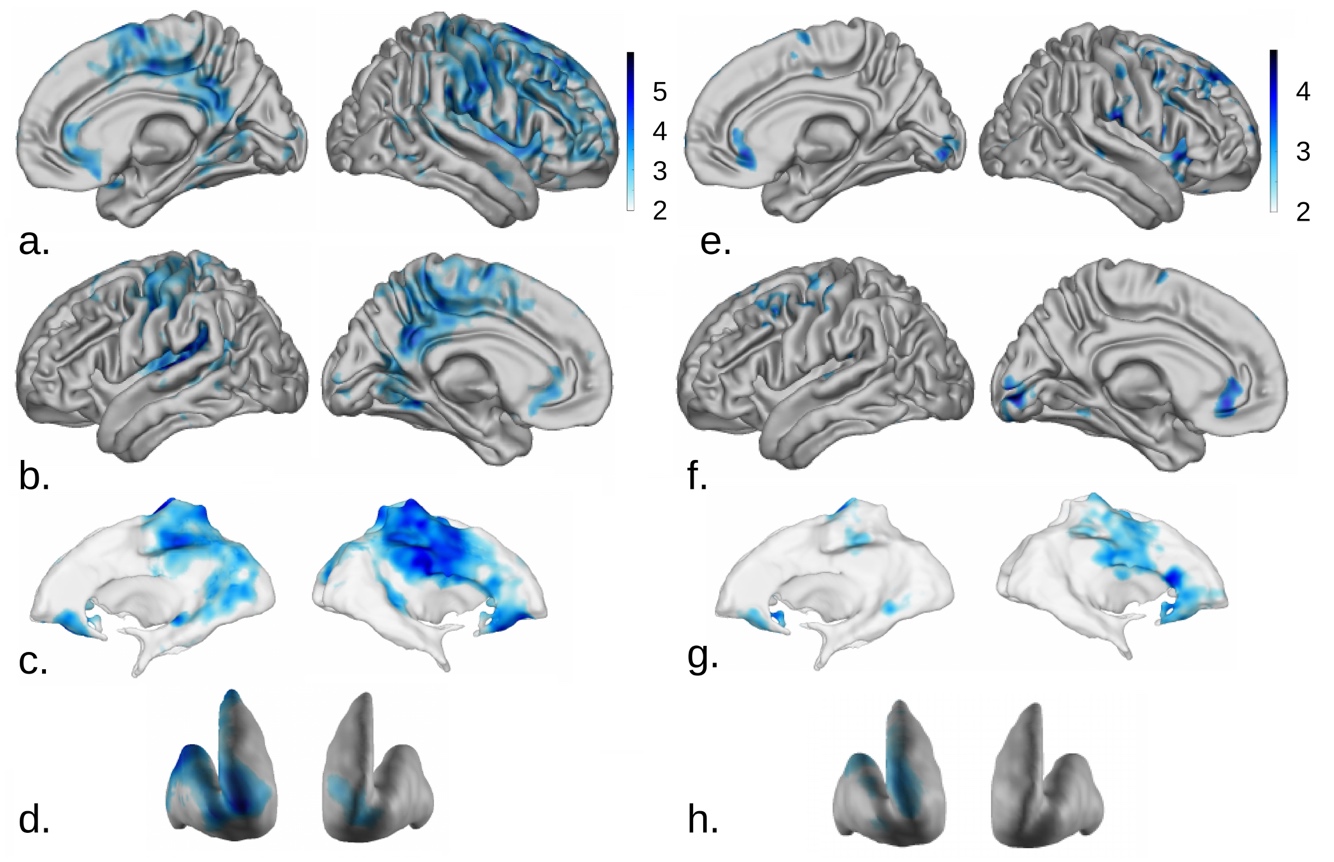 |
| --- |
| **Figure S3.** **Exposure to SED during childhood accounts for slowed down myelin marker growth more robustly than currently living in a poor neighbourhood. a.-d**. Reproduction of data in Figure 1 of main text for ease of comparison with e.-h. Areas shown where MT growth is slower when assessed longitudinally over visits (N=328/185 scans/subjects), as a function of reporting living in a deprived area before age 12. More explanations follow below. **e.-h.** As per a.-d., but the independent variable is deprivation status of the current neighbourhood of residence (current SED), on the same sample of participants as a.-d. Analysis of the entire N=479/288 scans/subjects sample strongly resembles e.-h (not shown). Rows show grey matter in right hemisphere, then left hemisphere, then core white matter, then striatal grey matter. Only the areas of Figure 1 Where current SED shows some significant associations at the lenient 15% FDR level are shown. All but 40 in this n=185 participant sample did not change their level of neighbourhood deprivation between before age 12 and testing; therefore we can say with some confidence that exposure during early development is associated with changes in MT increase (a.-d.) but the low economic mobility implies that the (lack of) impact of current deprivation has to be interpreted with great caution. |

###
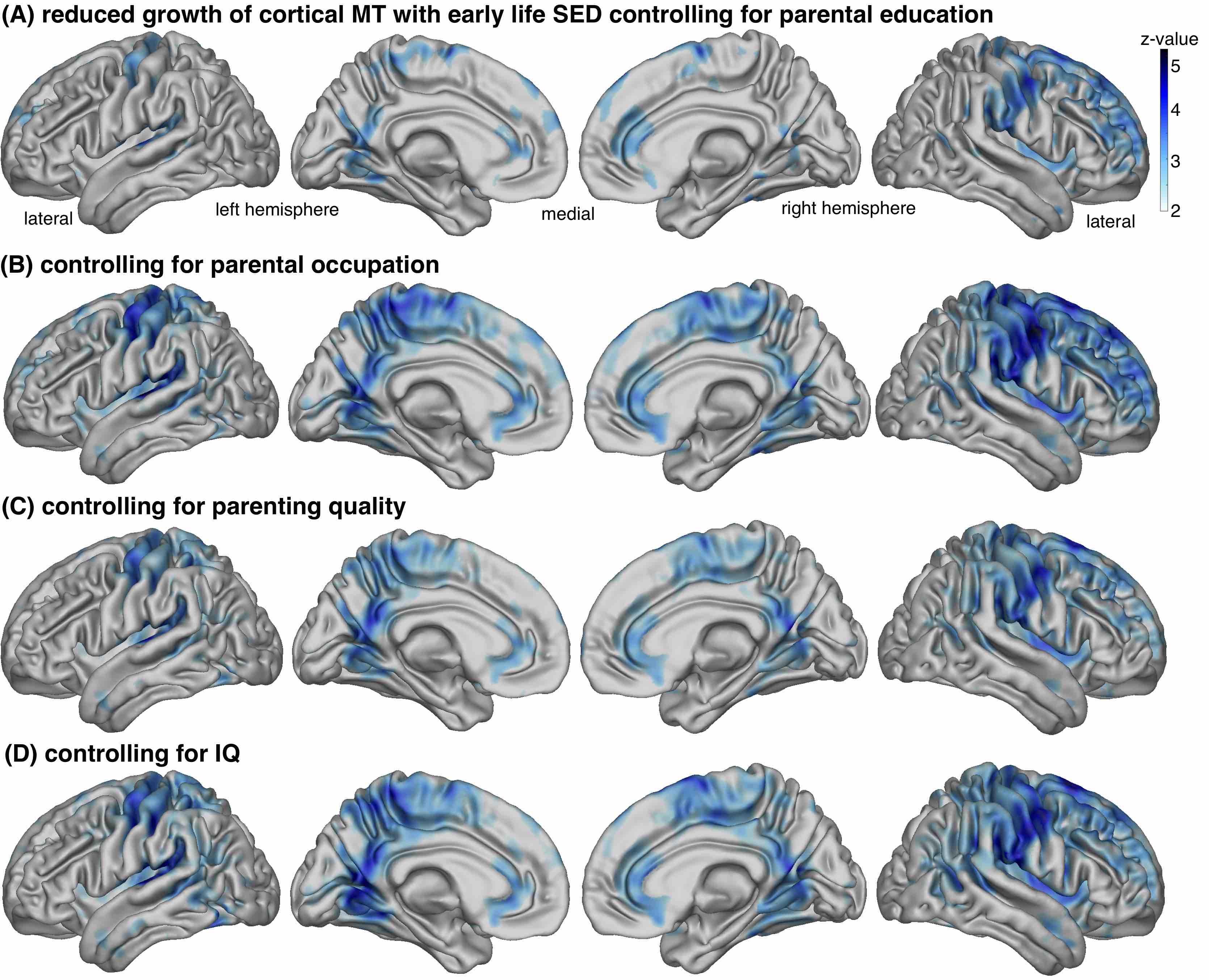


**Figure S4 Slower growth of myelin-sensitive MT as early life SED increases is partially explained by parental education but not other factors.** We present Z-maps showing negative SED by time/visit interactions, p<.05 FDR corrected, one-sided Wald tests, N=328/185 scans/subjects, 45.7% female, when additionally controlling for multiple covariates and their respective time/visit interactions in A-C). **A.** Controlling for parental education reduces the impact of SED, in medial motor and premotor areas more than right lateral prefrontal ones (cf. Figure S2a-d, very similar to (B) here) **B.** In contrast, controlling for parental occupation has minimal impact. **C.** Overall parenting quality has small impact (cf. main Figure 2). **D.** Controlling for time-varying IQ raw scores (here, WASI matrix) has negligible effect on the interaction (similar results for vocabulary, not shown). Controlling for baseline (or mean) IQ over the study period had a similar minimal effect. Colour scale is identical for A-D. Beta parameter (beta±95%CI) for reduced MT increase over visits with higher SED (in Figure 1D peak region central operculum/posterior insula at MNI [-52,-17,12] mm) without additional covariates (-0.0089±0.004), controlling for parental education (-0.0072±0.004), parental occupation (-0.0087±0.004), parenting quality (-0.0089±0.004), WASI matrix (-0.0089±0.004).

| **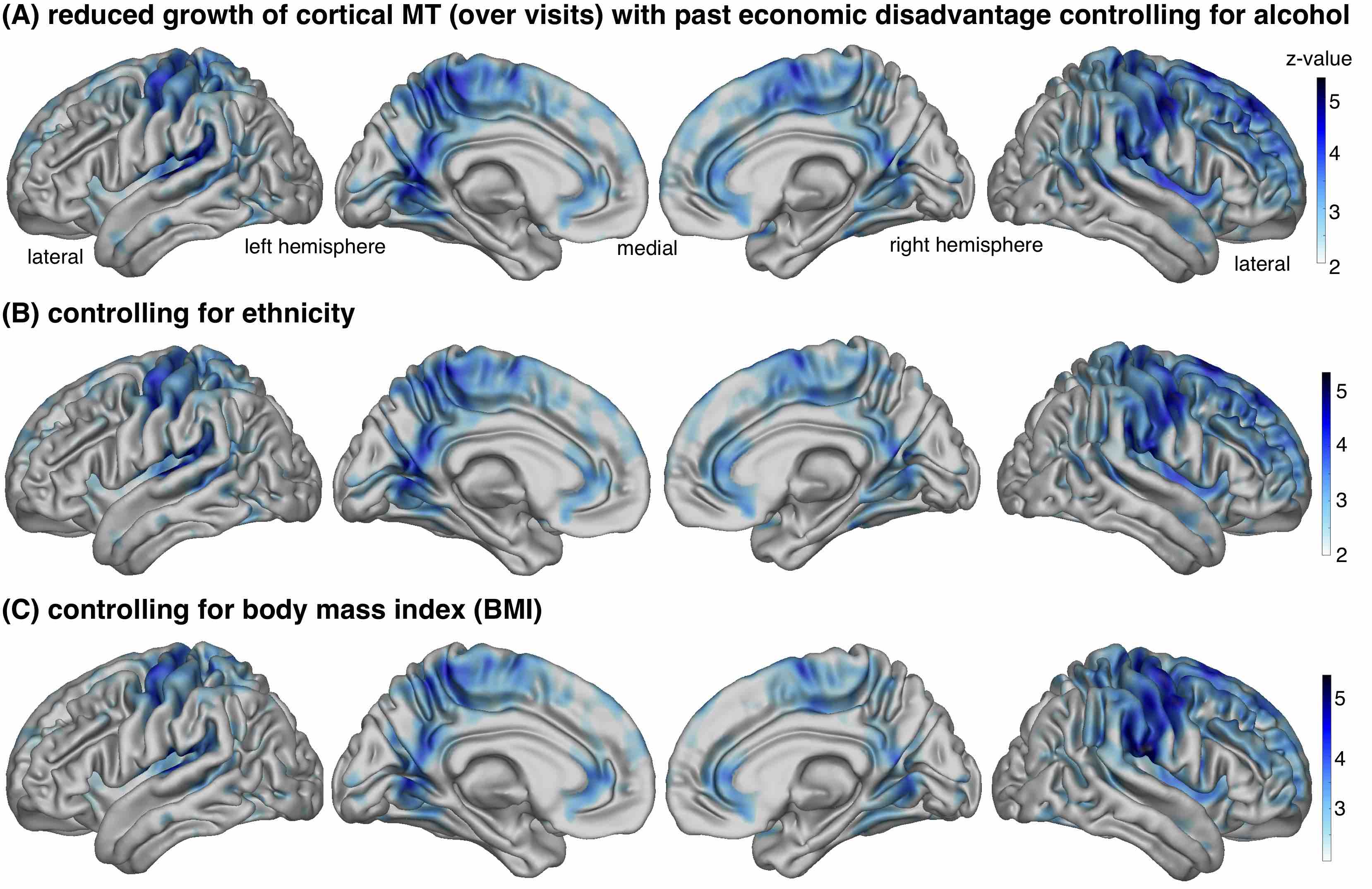** |
| --- |
| **Figure S5**. **Slower growth of myelin-sensitive MT as early life SED increases is not explained by alcohol, ethnicity or body mass index (BMI).** We present z-maps showing negative SED by time/visit interactions, p<.05 FDR corrected, N=328/185 scans/subjects, 45.7% female, when additionally controlling for multiple covariates and their respective time/visit interactions in A-C). |

| **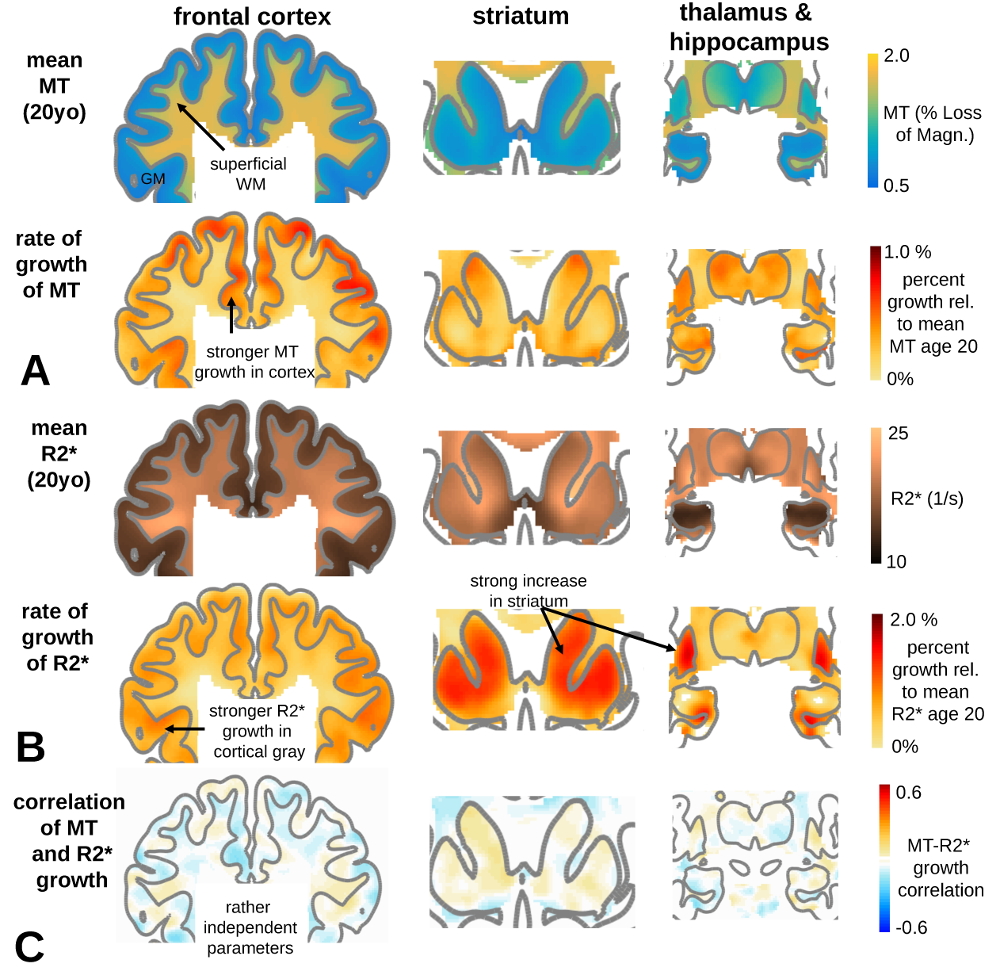** |
| --- |
| **Figure S6**. Comparison of MT and R2* multiparameter maps, showing that the areas where they show characteristic increases during the study period are largely independent. **A.** Top row shows that MT obtains highest values in white matter (yellow rather than blue) but bottom row shows that highest increases take place in cortical grey, dorsal striautm, and parts of the thalamus and hippocampus. **B.** Top row shows that R2* signal is strongest in cortical grey, hippocampus and ventral striatum, but bottom row shows strongest increases in the striatum. **C.** The amount of variance shared by the two measurements is negligible, with correlations around 0.1 . |

### D. Key morphometric measures

| 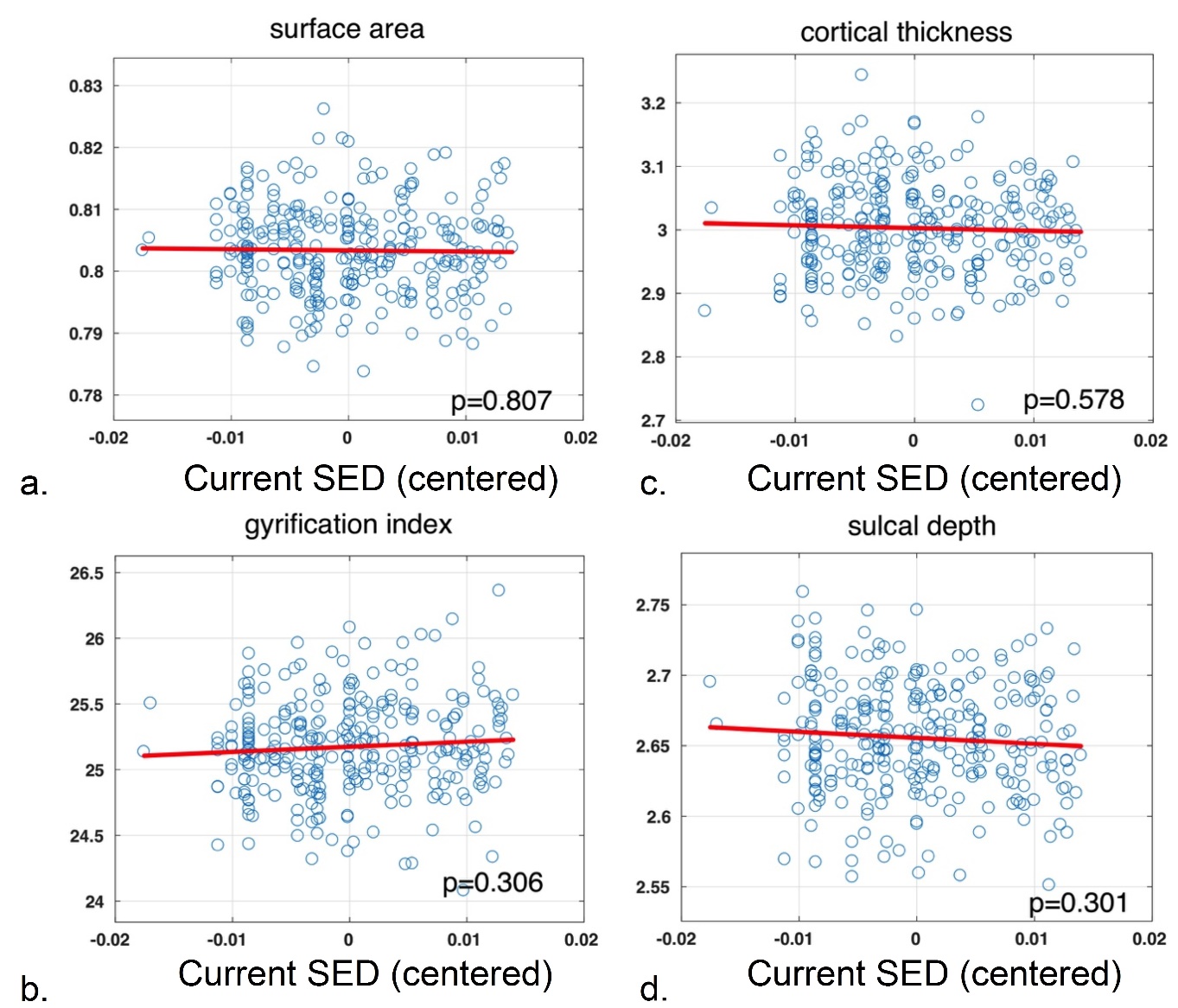 |
| --- |
| **Figure S7**. Morphometric measures, including some previously found to correlate with measures of SED different to the ones in our study, were not associated with socio-economic disadvantage. All global surface-based analyses shown here are cross-sectional and controlled for age, sex and their interaction. Linear fits are shown, but quadratic components were not significant either. **a.** to **d.** show representative analyses where global morphometric measures are plotted against current SED. Very similar, non-significant results were found when global morphometric measures were tested against early SED. Additionally, longitudinal analysis of local gray matter volume (GMV) in exact parallel to the main MT analyses showed no evidence for SED-related modulation of GMV shrinkage over visits. This suggests that MT saturation reveals a specific impairment of myelin growth trajectories. |

### D. Image map cluster statistics

**Supplementary Table S1. SPM results for negative early life SED by time/visit interaction on myelin-sensitive MT within grey matter**

| brain region | cluster size | p(FDR) voxelwise | Z | p(unc) | x | y | z |
| --- | --- | --- | --- | --- | --- | --- | --- |
| **cortical gray matter** |  |  |  |  |  |  |  |
| right posterior superior frontal gyrus | 367123 | 9.69E-04 | 5.47 | 2.2E-08 | 13 | 13 | 68 |
| left central operculum/insula |  | 9.69E-04 | 5.12 | 1.5E-07 | -52 | -17 | 12 |
| right central operculum/insula |  | 9.69E-04 | 5.03 | 2.4E-07 | 39 | 0 | 13 |
| right precentral gyrus |  | 9.69E-04 | 4.99 | 3.1E-07 | 51 | -6 | 49 |
| left medial postcentral gyrus |  | 9.69E-04 | 4.89 | 5.1E-07 | -9 | -43 | 64 |
| left superior parietal lobe |  | 9.69E-04 | 4.68 | 1.4E-06 | -35 | -49 | 52 |
| right postcentral gyrus |  | 9.69E-04 | 4.55 | 2.6E-06 | 22 | -32 | 71 |
| left precentral gyrus |  | 9.69E-04 | 4.53 | 3.0E-06 | -39 | -16 | 42 |
| right medial orbital gyrus | 1388 | 0.0029 | 3.59 | 1.6E-04 | 13 | 32 | -17 |
| right inferior occipital gyrus | 508 | 0.0064 | 3.16 | 7.9E-04 | 34 | -76 | 6 |
| left medial orbital gyrus | 535 | 0.012 | 2.77 | 0.003 | -19 | 12 | -15 |
| **subcortical and cerebellar  gray matter** |  |  |  |  |  |  |  |
| right inf. post. cereb. lobule VIIIB | 66522 | 0.003 | 5.45 | 2.5E-08 | 14 | -41 | -54 |
| left inf. post. cereb. lobule X |  | 0.003 | 4.64 | 1.7E-06 | -16 | -38 | -45 |
| left inf. post. cereb. lobule VIIIA |  | 0.003 | 4.62 | 1.9E-06 | -18 | -61 | -56 |
| right ant. cereb. lobule III |  | 0.003 | 4.43 | 4.7E-06 | 5 | -42 | -12 |
| left sup. post. cereb. lobule VI |  | 0.003 | 4.19 | 1.4E-05 | -22 | -54 | -28 |
| left sup. post. cereb. lobule crus II |  | 0.003 | 4.16 | 1.6E-05 | -51 | -51 | -43 |
| right ant. cereb. lobule IV |  | 0.004 | 3.95 | 3.9E-05 | 24 | -31 | -26 |
| right inf. post. cereb. lobule VIIIA |  | 0.004 | 3.84 | 6.1E-05 | 39 | -51 | -56 |
| right ant. caudate | 4987 | 0.004 | 3.78 | 7.9E-05 | 16 | 28 | -4 |
| right putamen |  | 0.005 | 3.75 | 8.7E-05 | 29 | -1 | 14 |
| left ant. caudate | 3445 | 0.006 | 3.56 | 0.0034 | -18 | 24 | 2 |
| right post. hippocampus | 2263 | 0.009 | 3.15 | 8.1E-04 | 27 | -31 | -4 |
| left post. hippocampus | 732 | 0.012 | 2.95 | 0.0016 | -29 | -40 | -3 |

[*table legend continued overleaf* ]

SPM longitudinal SwE results table testing for negative early life SED by time/visit interaction effects on MT in cortical and subcortical grey matter accounting for covariates and confounds (cf. methods and supplementary notes on modelling, n=328/185 scans/subjects). Voxel resolution 1mm isotropic. Voxelwise FDR corrected (p<0.05)

reporting peaks and clusters with up to 8 local maxima more than 24 mm apart, applied extent threshold k=500 voxel. We report FDR on whole-brain level. Using SwE covariance type ‘classic’, effective degrees of freedom per subject were estimated as 0.973.

**Supplementary Table S2. SPM results for negative early life SED by time/visit interaction on myelin-sensitive MT within white matter**SPM longitudinal SwE results table testing for negative early life SED by time/visit interaction effects on MT in white matter accounting for covariates and confounds (cf. methods and supplementary notes on modelling, n=328/185 scans/subjects). Voxel resolution 1mm isotropic. Voxelwise FDR corrected (p<0.05) reporting peaks and clusters with up to 4 local maxima more than 24 mm apart, applied extent threshold k=500 voxel. We report FDR on whole-brain level. Using SwE covariance type ‘classic’, effective degrees of freedom per subject were estimated as 0.973.

| Brain region | cluster size | p(FDR) voxelwise | Z | p(unc) | x | y | z |
| --- | --- | --- | --- | --- | --- | --- | --- |
| **cortex-adjacent white matter** |  |  |  |  |  |  |  |
| right postcentral gyrus | 73047 | 0.014 | 4.40 | 5.5E-06 | 47 | -14 | 37 |
| right medial precentral gyrus |  | 0.014 | 4.25 | 1.1E-05 | 15 | -10 | 46 |
| right supplementary motor area |  | 0.014 | 4.18 | 1.5E-05 | 7 | -2 | 68 |
| right medial orbital gyrus |  | 0.014 | 3.95 | 3.9E-05 | 19 | 34 | -7 |
| left precuneus | 39333 | 0.014 | 4.01 | 3.0E-05 | -5 | -58 | 57 |
| left superior postcentral gyrus |  | 0.014 | 3.95 | 4.0E-05 | -17 | -41 | 70 |
| left inf. postcentral gyrus |  | 0.014 | 3.86 | 5.6E-05 | -47 | -23 | 46 |
| left sup. temporal gyrus |  | 0.014 | 3.64 | 1.3E-04 | -56 | -40 | 17 |
| right precuneus | 1407 | 0.014 | 3.86 | 5.6E-05 | 5 | -55 | 17 |
| right ant. middle frontal gyrus | 660 | 0.014 | 3.78 | 7.7E-05 | 36 | 57 | 9 |
| right post. middle frontal gyrus |  | 0.014 | 3.31 | 4.7E-04 | 35 | 38 | 35 |
| left post. cingulate gyrus | 2556 | 0.014 | 3.77 | 8.0E-05 | -5 | -49 | 26 |
| left lingual gyrus | 715 | 0.014 | 3.54 | 2.0E-04 | -10 | -60 | 4 |
| right ant. sup. frontal gyrus | 1357 | 0.014 | 3.53 | 2.1E-04 | 24 | 62 | 12 |
| right post. sup. frontal gyrus |  | 0.015 | 2.98 | 0.0015 | 7 | 47 | 45 |
| right lingual gyrus | 756 | 0.018 | 2.84 | 0.0023 | 7 | -61 | 6 |
| white matter core areas |  |  |  |  |  |  |  |
| rigth ant. corona radiata | 26531 | 0.01 | 3.98 | 3.4E-05 | 18 | 33 | -5 |
| right sup. corona radiata |  | 0.01 | 3.94 | 4.1E-05 | 19 | -12 | 44 |
| right external capsule |  | 0.01 | 3.87 | 5.5E-05 | 31 | 2 | 10 |
| left post. thalamic radiation | 4465 | 0.011 | 3.16 | 7.8E-04 | -41 | -42 | 2 |
| left sup. longitudinal fascic. | 1527 | 0.013 | 2.98 | 0.0014 | -38 | -24 | 28 |

**Supplementary Table S3. SPM results for early life SED by parenting quality by time/visit interaction on cortical myelin-sensitive MT**

| Brain region | cluster size | p(FDR) voxelwise | Z | p(unc) | x | y | z |
| --- | --- | --- | --- | --- | --- | --- | --- |
| **cortical gray matter** |  |  |  |  |  |  |  |
| right ant. middle frontal gyrus | 5327 | 2.9E-04 | 5.69 | 6.5E-09 | 29 | 55 | -3 |
| right ant. sup. frontal gyrus |  | 0.011 | 4.08 | 2.2E-05 | 27 | 52 | 36 |
| left inf. frontal angular gyrus | 10028 | 0.006 | 4.33 | 7.4E-06 | -49 | 33 | -8 |
| left ant. middle frontal gyrus |  | 0.007 | 4.27 | 9.6E-06 | -43 | 49 | 19 |
| left inf. frontal gyrus |  | 0.007 | 4.25 | 1.0E-05 | -54 | 25 | 20 |
| left ant. middle frontal gyrus |  | 0.018 | 3.77 | 8.3E-05 | -28 | 56 | -3 |
| right ant. middle frontal gyrus | 426 | 0.007 | 4.24 | 1.1E-05 | 34 | 52 | 11 |
| left medial superior frontal gyrus | 335 | 0.013 | 4.00 | 3.1E-05 | -6 | 55 | 30 |
| right post. middle frontal gyrus | 413 | 0.021 | 3.70 | 1.1E-04 | 36 | 8 | 55 |
| left sup. frontal gyrus | 301 | 0.023 | 3.61 | 1.5E-04 | -16 | 28 | 55 |
| **cortex-adjacent white matter** |  |  |  |  |  |  |  |
| right ant. middle frontal gyrus | 588 | 0.017 | 4.65 | 1.6E-06 | 32 | 56 | -2 |
| right sup. frontal gyrus | 295 | 0.017 | 4.44 | 4.4E-06 | 14 | 60 | -8 |

SPM longitudinal SwE results table testing for positive early life SED by parenting quality by time/visit interaction effects on MT in cortical grey and adjacent white matter accounting for covariates and confounds (cf. methods and supplementary notes on modelling, n=328/185 scans/subjects). Voxel resolution 1mm isotropic. Voxelwise FDR corrected (p<0.05) reporting peaks and clusters with up to 4 local maxima more than 24 mm apart, applied extent threshold k=250 voxel. Using SwE covariance type ‘classic’, effective degrees of freedom per subject were estimated as 0.9568.

**Supplementary Table S4. SPM results for parenting quality by sex interaction on cortical myelin-sensitive MT**

| Brain region | cluster size | p(FDR) voxelwise | Z | p(unc) | x | y | z |
| --- | --- | --- | --- | --- | --- | --- | --- |
| **cortical gray matter** |  |  |  |  |  |  |  |
| right precuneus | 94498 | 0.003 | 5.30 | 5.7E-08 | 7 | -52 | 23 |
| right superior parietal lobe |  | 0.003 | 5.14 | 1.3E-07 | 31 | -53 | 48 |
| right post. cingulate gyrus |  | 0.003 | 5.12 | 1.5E-07 | 3 | -38 | 45 |
| left precentral gyrus | 7290 | 0.003 | 5.10 | 1.7E-07 | -41 | -12 | 32 |
| left postcentral gyrus |  | 0.004 | 4.05 | 2.6E-05 | -24 | -28 | 56 |
| right precentral gyrus | 15031 | 0.003 | 5.03 | 2.4E-07 | 34 | -15 | 43 |
| right postcentral gyrus |  | 0.003 | 4.21 | 1.3E-05 | 27 | -36 | 53 |
| right supramarginal gyrus |  | 0.004 | 4.07 | 2.4E-05 | 60 | -18 | 42 |
| left inf. temporal gyrus | 4774 | 0.003 | 4.62 | 1.9E-06 | -53 | -47 | -26 |
| left middle temporal gyrus |  | 0.006 | 3.77 | 8.2E-05 | -47 | 3 | -27 |
| left middle temporal gyrus |  | 0.008 | 3.56 | 1.9E-04 | -60 | -25 | -10 |
| left post. superior frontal gyrus | 3450 | 0.003 | 4.55 | 2.7E-06 | -21 | -10 | 58 |
| left middle frontal gyrus |  | 0.009 | 3.40 | 3.3E-04 | -26 | 14 | 53 |
| right central operculum | 4475 | 0.003 | 4.30 | 8.7E-06 | 42 | -10 | 17 |
| right parietal operculum |  | 0.005 | 3.82 | 6.6E-05 | 57 | -28 | 23 |
| left medial orbital gyrus | 3470 | 0.003 | 4.22 | 1.2E-05 | -24 | 31 | -15 |
| right post. gyrus rectus | 6722 | 0.004 | 4.10 | 2.1E-05 | 6 | 25 | -22 |
| right ant. gyrus rectus | 3600 | 0.007 | 3.67 | 1.2E-04 | 3 | 62 | -22 |

SPM longitudinal SwE results table testing for positive parenting quality by sex interaction effects on MT in cortical grey matter accounting for covariates and confounds (cf. methods and supplementary notes on modelling, n=328/185 scans/subjects). Voxel resolution 1mm isotropic. Voxelwise FDR corrected (p<0.05) reporting peaks and clusters with up to 3 local maxima more than 24 mm apart, applied extent threshold k=3000 voxel. We report FDR on whole-brain level. Using SwE covariance type ‘classic’, effective degrees of freedom per subject were estimated as 0.9568.

**Supplementary Table S5. SPM results for body mass index effects on cortical myelin-sensitive MT**

| Brain region | cluster size | p(FDR) voxelwise | Z | p(unc) | x | y | z |
| --- | --- | --- | --- | --- | --- | --- | --- |
| **cortical gray matter** |  |  |  |  |  |  |  |
| right frontal operculum/insula | 447 | 0.021 | 5.06 | 2.1E-07 | 29 | 23 | 13 |
| left superior temporal gyrus | 385 | 0.021 | 4.72 | 1.2E-06 | -48 | -4 | -16 |
| right subgenual gyrus | 1256 | 0.021 | 4.54 | 2.8E-06 | 9 | 37 | -3 |
| right ant. midcingulate gyrus |  | 0.029 | 4.04 | 2.7E-05 | 2 | 8 | 44 |
| right ant. cingulate gyrus |  | 0.034 | 3.89 | 5.0E-05 | 4 | 27 | 24 |
| left sup. frontal gyrus | 250 | 0.027 | 4.08 | 2.2E-05 | -24 | -9 | 54 |

SPM longitudinal SwE results table testing for negative body mass index effects on MT in cortical grey matter accounting for covariates and confounds (cf. methods and supplementary notes on modelling, n=277/155 scans/subjects). Voxel resolution 1mm isotropic. Voxelwise FDR corrected (p<0.05) reporting peaks and clusters with up to 3 local maxima more than 24 mm apart, applied extent threshold k=250 voxel. We report FDR on whole-brain level. Using SwE covariance type ‘classic’, effective degrees of freedom per subject were estimated as 0.9484.

**Supplementary Table S6. Dependence of global MT and R2* measures on development and on socio-economic disadvantage**

|  | Model: Y ~ sex + β1 age/visit + β2 SED + β3 age/visit by SED | | | | |  |
| --- | --- | --- | --- | --- | --- | --- |
| Anatomical Metric | β1 (SE) | p_β1 | β2 (SE) | p_β2 | β3 (SE) | p_β3 |
| Gray matter volume (cubic cm) | -7.99 (0.89) | 2.4e-17 | -0.16  (0.59) | 0.79 | -0.0264 (0.12) | 0.83 |
| White matter volume (cubic cm) | 0.17 (0.67) | 0.80 | 0.12  (0.52) | 0.82 | -0.0047 (0.09) | 0.96 |
| Total surface area (square cm) | -10.02 (2.3) | 1.9e-5 | -0.72  (1.66) | 0.66 | -0.12 (0.32) | 0.72 |
| Mean cortical thickness (mm) | -0.014 (0.0022) | 1.6e-9 | 0.0014 (0.00095) | 0.15 | -0.00032 (0.00029) | 0.27 |
| Hippocampal volume (cubic cm) | -0.0044 (0.0046) | 0.33 | 0.0033 (0.0028) | 0.25 | -0.0001 (0.0006) | 0.86 |
| Mean cortical MT sat (% loss. of Magn.) | 0.006 (0.0009) | 2.1e-10 | 3e-5  (0.0002) | 0.89 | -0.00024 (0.00012) | 0.037 |
| Mean GM MT sat (% loss. of Magn.) | 0.0056 (0.0008) | 3.1e-11 | -0.0004 (0.0002) | 0.026 | -0.00024 (0.0001) | 0.022 |
| Mean WM MT sat (% loss. of Magn.) | 0.0058 (0.0016) | 0.00045 | -0.00038 (0.0006) | 0.55 | -0.0006 (0.0002) | 0.0059 |
| Mean GM R2s (1/s) | 0.13 (0.0097) | 7e-35 | -0.0033 (0.0040) | 0.41 | -0.0023 (0.0013) | 0.074 |
| Mean WM R2s (1/s) | 0.12 (0.011) | 8e-23 | 0.0035 (0.0067) | 0.60 | -0.00045 (0.0015) | 0.76 |

Linear mixed effects regression of MT saturation for global cortical, grey matter and white matter segmentations show a significant interaction term with age/visit, consistent with main analyses (yellow). However, a similar analysis for R2* does not reach significance, supporting an interpretation of MT as a measurement of myelin, rather than non-myelin variance shared with R2* (grey). Model contains intercept as random effects, centered sex and age/visit as fixed effects. Contrast estimates (SE) of main effect of age/visit, SED and SED by age/visit interactions are shown.

### E. Explanatory note on pleiotropy

| 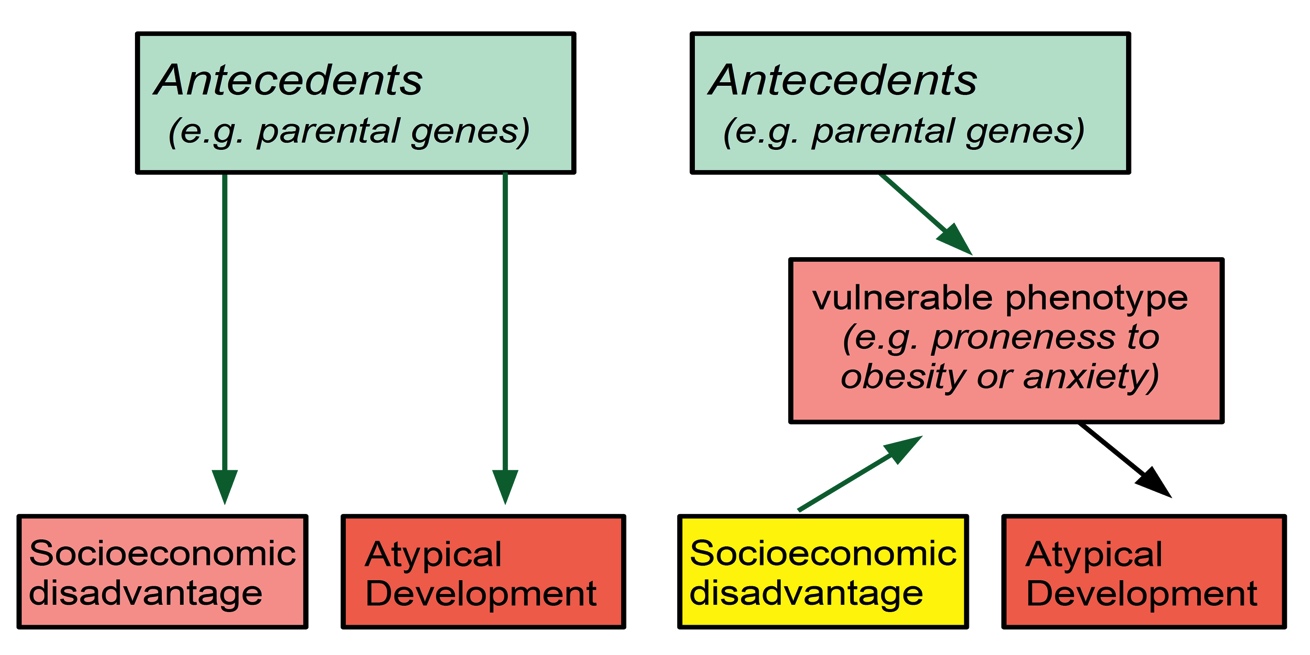 **a.**  **b** |
| --- |
| **Figure S8.** **Psycho-biological approaches to SED-induced effects on brain growth.** **(a)** Horizontal pleiotropy: An early antecedent causes atypical development side-by-side with another phenotype (here, SED). Hypothetically, genes conferring suboptimal neurocognition in the parents cause them to fail economically, while independently and deterministically give rise to atypical myelination in their offspring. **(b)** Vertical pleiotropy: An antecedent gives rise to a 'vulnerable phenotype', which precedes the second, observed phenotype (in our example, atypical myelination). SED amplifies progression through this vertical cascade, whereas a health-promoting socio-economic status would block it. Hypothetically, genes conferring a ‘sweet tooth’, or high neuroticism may affect neurodevelopment only through obesity, or chronic anxiety. However, the cascade is realized only if SED contributes to poor nutrition, or chronic stress. Horizontal pleiotropy is too restrictive in the light of our results, but vertical pleiotropy is more likely. Vertical and horizontal pleiotropy usually refer to genetic early antecedents, but in our case other early environmental insults might also initiate vulnerable phenotypes. |

### F. Neuroscience in Psychiatry consortium

**Supplementary table 6. Neuroscience in Psychiatry consortium author list.**

| **Principal investigators:** |
| --- |
| Edward Bullmore (CI from 01/01/2017) |
| Raymond Dolan |
| Ian Goodyer (CI until 01/01/2017) |
| Peter Fonagy |
| Peter Jones |
| **NSPN (funded) staff:** |
| Michael Moutoussis |
| Tobias Hauser |
| Sharon Neufeld |
| Rafael Romero-Garcia |
| Michelle St Clair |
| Petra Vértes |
| Kirstie Whitaker |
| Becky Inkster |
| Gita Prabhu |
| Cinly Ooi |
| Umar Toseeb |
| Barry Widmer |
| Junaid Bhatti |
| Laura Villis |
| Ayesha Alrumaithi |
| Sarah Birt |
| Aislinn Bowler |
| Kalia Cleridou |
| Hina Dadabhoy |
| Emma Davies |
| Ashlyn Firkins |
| Sian Granville |
| Elizabeth Harding |
| Alexandra Hopkins |
| Daniel Isaacs |
| Janchai King |
| Danae Kokorikou |
| Christina Maurice |
| Cleo McIntosh |
| Jessica Memarzia |
| Harriet Mills |
| Ciara O’Donnell |
| Sara Pantaleone |
| Jenny Scott |
| **Affiliated scientists:** |
| Pasco Fearon |
| John Suckling |
| Anne-Laura van Harmelen |
| Rogier Kievit |
